# Engineered probiotics platform for resolvin E1 biosynthesis confers protection against inflammatory disease

**DOI:** 10.1002/ctm2.70746

**Published:** 2026-07-19

**Authors:** Xiaoxiao Li, Jiejing Lin, Qingqing Liu, Yiqun Wu, Qian Wu, Zewei Zhao, Yi Cai, Shengliang Lin, Zijie Zheng, Xinpan Chen, Jin Li, Zhonghan Yang

**Affiliations:** ^1^ Shenzhen Key Laboratory for Systems Medicine in Inflammatory Diseases & Department of Biochemistry Zhongshan School of Medicine Sun Yat‐sen University Shenzhen Campus Shenzhen China; ^2^ Department of Geriatrics The First Affiliated Hospital Sun Yat‐sen University Guangzhou China

**Keywords:** engineered probiotics, *Escherichia coli* Nissle 1917, gut microbiota, inflammation resolution, resolvin E1

## Abstract

**Background:**

The resolution of inflammation is actively driven by omega‐3 polyunsaturated fatty acids (PUFAs) via their specialized pro‐resolving mediator (SPM) derivatives, including resolvin E1 (RvE1), whose role has been well established. However, clinical application of these mediators is hampered by inherent instability and elevated production costs.

**Methods:**

To surmount these obstacles, we have engineered a biosynthetic platform based on the probiotic *Escherichia coli* Nissle 1917 (EcN) that enables controlled, sustained RvE1 production through inducible expression of COX2 and 5‐LOX, designated EcN‐RvE1. The catalytic capacity, intestinal persistence, and therapeutic efficacy of the platform were evaluated in vitro and in LPS‐induced acute inflammation and DSS‐induced colitis murine models.

**Results:**

In this study, we validate the capacity of EcN‐RvE1 to catalyse the conversion of eicosapentaenoic acid (EPA) to RvE1 and confirm its ability to achieve long‐term intestinal persistence. In murine models of acute inflammation and colitis, EcN‐RvE1 exerts marked anti‐inflammatory and tissue‐protective effects, which are mediated by the regulation of inflammatory cytokine expression and the amelioration of gut microbiota dysbiosis. Moreover, EcN‐RvE1 using *Euglena gracilis* as a photosynthetic protist‐based source of PUFAs also exhibits protective anti‐inflammatory activity.

**Conclusion:**

Collectively, we report a probiotic engineering platform for the biosynthesis of RvE1, offering a novel strategy for harnessing the anti‐inflammatory potential of PUFAs derivatives in clinical settings.

## Highlights

- Engineered EcN‐RvE1 enables inducible, sustained RvE1 biosynthesis from EPA
- EcN‐RvE1 achieves long‐term intestinal persistence through oral administration
- EcN‐RvE1 alleviates LPS‐induced acute systemic inflammation and DSS‐induced colitis in mice
- EcN‐RvE1 works synergistically with *Euglena gracilis* as a synbiotic formulation

## Graphical abstract

An engineered EcN‐RvE1 probiotic platform enables sustained biosynthesis of the pro‐resolving mediator RvE1 via inducible COX2/5‐LOX expression. Oral administration achieves long‐term intestinal persistence and systemic anti‐inflammatory effects in murine models of acute inflammation and colitis, offering a promising oral therapeutic strategy for inflammatory diseases.

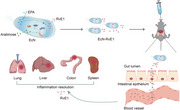

## 1 INTRODUCTION

The management of both acute and chronic inflammation remains a major clinical challenge, despite extensive research in related fields.^1^, ^2^ Acute inflammation, triggered by infection or tissue injury, can rapidly escalate into life‐threatening systemic responses if uncontrolled.^3^ Although the use of glucocorticoids and biologic agents can potently suppress inflammation, these approaches cause significant side effects and fail to reverse established tissue damage.^4^, ^5^ In contrast, persistent acute inflammation can give rise to chronic conditions including inflammatory bowel disease (IBD), systemic lupus erythematosus, and atherosclerosis, leading to sustained tissue injury.^6^, ^7^ Accordingly, therapeutic strategies that actively promote the resolution of inflammation, rather than merely suppressing it, are urgently needed.^8^, ^9^

Specialized pro‐resolving mediators (SPMs) are endogenous lipids mediators derived from polyunsaturated fatty acids (PUFAs) that actively orchestrate the resolution of inflammation.^10^, ^11^ In contrast to the conventional anti‐inflammatory drugs, SPMs inhibit pro‐inflammatory signalling, limit neutrophil infiltration, modulating macrophage polarization,^12^, ^13^ and stimulate tissue repair without compromising host defense.^14^ The SPMs family includes lipoxins (LXs),^15^ resolvins (Rvs),^16^, ^17^ protectins (PDs) and maresins (MaRs).^18^ Among them, resolvin E1 (RvE1), derived from the ω‐3 PUFA eicosapentaenoic acid (EPA), exerts potent pro‐resolving actions by interacting with the G protein‐coupled receptor ChemR23 (CMKLR1)^19^, ^20^ and antagonizing the leukotriene B4 receptor BLT1.^21^ RvE1 has demonstrated therapeutic potential in diseases such as colitis,^22^ periodontitis,^23^ acute lung injury,^24^ viral infection^25^ and atherosclerosis.^26^

Nevertheless, the clinical translation of RvE1 remains challenging. As an endogenous lipid mediator, RvE1 is chemically unstable, resulting in an extremely short half‐life in vivo.^10^, ^11^ Furthermore, the high production costs associated with cumbersome total chemical synthesis^27^, ^28^ and the necessitation for targeted delivery to inflamed tissues^29^ also regarded as major bottlenecks. These limitations call for alternative strategies that can produce RvE1 sustainably and locally.

Recent advances in synthetic biology offer a promising alternative: engineering non‐pathogenic probiotic bacteria to produce therapeutic molecules in situ of the gut.^30^, ^31^ The probiotic *Escherichia coli* Nissle 1917 (EcN) has a well‐documented safety profile and is amenable to genetic modification for biosynthesize of complex bioactive compounds.^32^, ^33^ Notably, engineered *Escherichia coli* has been successfully used for oral delivery of antibodies and the regulation of cysteine metabolism.^34^, ^35^ In this study, we established an EcN‐based platform, designated EcN‐RvE1, that enables sustained biosynthesis of RvE1 from EPA via an arabinose‐inducible tandem human cyclooxygenase 2 (COX2) and 5‑lipoxygenase (5‑LOX) expression cassette. Our results demonstrated that oral administration of EcN‐RvE1 achieves long‐term intestinal persistence ability, exhibited significant inflammation‐alleviative and tissue‐protective effects in murine models of acute inflammation and colitis. Furthermore, we show that the photosynthetic protist‐based omega‑3 source *Euglena gracilis* can replace pure EPA as a sustainable substrate, suggesting a potential synbiotic formulation. Collectively, our work provides a novel, non‑invasive and cost‑effective strategy for the development of inflammation‐directed SPMs‑based therapies to overcome the translational bottlenecks.

## 2 Materials and methods

### 2.1 Bacteria strains and plasmid construction

*Escherichia coli* Nissle 1917 (EcN) obtained from Shanghai Weidi Biotechnology (China) was used as the chassis probiotic strain. The coding sequences of human COX2 (PTGS2) and 5‐LOX (ALOX5) were codon‐optimized for prokaryotic expression (Table S1) and synthesized by Guangzhou Ruibo Biotechnology Co., Ltd. The P2A self‐cleaving peptide was used to enable equimolar co‐expression of COX2 and 5‐LOX from a single transcript. The arabinose‐inducible pBAD vector was used for all constructions. For single‐enzyme expression, COX2 or 5‐LOX genes was amplified by Phanta^®^ Max Master Mix (Cat# P515‐02, Vazyme, China) and cloned into pBAD vectors, generating pBAD‐COX2 and pBAD‐5‐LOX. All primers used for cloning were listed in Table S1. The recombinant plasmids were extracted using EndoFree Mini Plasmid Kit II (Cat# DP118‐02, TIANGEN BIOTECH, China), validated by Sanger sequencing (Beijing Tsingke Biotech Co., Ltd.), and transformed into EcN via heat shock method, yielding strains EcN‐COX2, EcN‐5‐LOX and EcN‐COX2‐P2A‐5‐LOX (designated EcN‐RvE1). All strains were grown at 37°C in Luria‐Bertani (LB) medium supplemented with ampicillin (50 µg/mL).

### 2.2 Induction of protein expression and Western blot analysis

Overnight cultures of recombinant EcN strains were diluted 1:100 in fresh LB medium containing ampicillin and grown at 37°C to an OD_600_ of 0.6. Arabinose was added to a final concentration of 0.4% to induce expression, and the cultures were incubated for an additional 12 h at either 30°C or 37°C. Bacterial pellets were extracted using RIPA buffer (Cat# CW2333S, CWBIO, China) supplemented with protease inhibitors (Cat# CW2200S, CWBIO, China). 20 µL of protein was separated by SDS‐PAGE and transferred onto 0.45 µm nitrocellulose (NC) membranes (Cat# HATF00010, Sigma‐Aldrich, USA) and blocked with 5% non‐fat milk in PBS‐T for 1 hour at room temperature. Membranes were incubated overnight at 4°C with primary antibodies against Cyclooxygenase 2 rabbit (Cat# R23971, ZEN‐BIOSCIENCE, China), 5 Lipoxygenase (Cat# R382216, ZEN‐BIOSCIENCE, China) and RecA (Cat# HY‐P87059, MCE, USA), followed by IRDye® 800CW‐conjugated secondary antibody (Cat# 926‐32211, Li‐COR Biosciences, USA). Protein bands were visualized using ChemiDoc MP Imaging System (Bio‐Rad, USA).

### 2.3 RvE1 biosynthesis and quantification in vitro

For co‐culture assays, EcN‐COX2 and EcN‐5‐LOX were grown separately to an OD_600_ of 1.0, then mixed at equal cell densities. EcN‐COX2‐P2A‐5‐LOX was grown to the same OD_600_. All cultures were induced with 0.4% arabinose for 12 h at 37°C. Aspirin (Cat# A800349, Macklin, China) was added to a final concentration of 2 mM to acetylate COX2, and 0.1% (v/v) EPA (Cat# Q114646, Dibo, China) was added as the substrate. After 8 h of further incubation, culture supernatants were collected by centrifugation. RvE1 concentration was measured using an ELISA kit (Cat# EK11191, Signalway Antibody, USA) according to the manufacturer's instructions. All samples were assayed in duplicate and concentrations were calculated against a four‐parameter logistic standard curve. Detailed methods are provided in Supplementary Data 1.

### 2.4 Bacterial Growth Curve Analysis

Overnight cultures of recombinant EcN strains were diluted 1:100 in fresh LB medium containing appropriate antibiotics and grown at 37°C with shaking (200 rpm). Bacterial growth was monitored by measuring OD_600_ at 2‐hour intervals over 36 hours.

### 2.5 Oxylipin analysis by mass spectrometry

Culture supernatants from EcN‐RvE1 induced with 0.4% arabinose, ASA, and EPA (or vehicle control) were subjected to oxylipin profiling. Eicosanoids contents were detected by MetWare (http://www.metware.cn/) based on the AB Sciex QTRAP 6500 LC‐MS/MS platform. Detailed methods are provided in Supplementary Data 1.

### 2.6 Animal models and care

All animal experiments were approved by the Institutional Animal Care and Use Committee of Sun Yat‐sen University (approval number: SYSU‐IACUC‐MED‐2023‐B113). 8‐week‐old female C57BL/6 mice purchased from the Center of Laboratory Animal at Sun Yat‐sen University were used in all models.

### 2.7 LPS‐induced acute inflammation model

C57BL/6 mice were randomly divided into seven groups: (1) Blank control (no treatment); (2) PBS vehicle control (intragastric gavage with PBS containing 0.4% Ara); (3) EcN control (10^9^ CFUs EcN with 0.4% Ara); (4) ASA alone; (5) EPA alone; (6) EcN‐RvE1 (10^9^ CFUs EcN‐RvE1 with 0.4% Ara); and (7) EcN‐RvE1‐bio (10^9^ CFUs EcN‐RvE1 supplemented with ASA and EPA with 0.4% Ara). Mice in groups 2‐7 received i.g. administration three times weekly (days 1, 3, and 6). Aspirin was freshly added to the bacterial suspension at a final concentration of 2 mM immediately before intragastric gavage, matching the concentration used in the in vitro assay. 48 hours after the final gavage, mice except the blank control group were challenged with LPS (5 mg/kg, i.p.). Mice was euthanized 6 h later for blood and tissue samples collection.

### 2.8 DSS‐induced colitis model

Colitis was induced by administering 4% (w/v) DSS in drinking water for 7 consecutive days. C57BL/6 mice were randomly divided into eight groups: G1 (control, EPA without DSS), G2 (EPA + DSS), G3 (EcN + EPA without DSS), G4 (EcN + EPA + DSS), G5 (EcN‐RvE1 + euglena without DSS), G6 (EcN‐RvE1 + euglena + DSS), G7 (EcN‐RvE1 + EPA without DSS) and G8 (EcN‐RvE1 + EPA + DSS). Treatments were administered i.g. three times weekly, with 0.4% Ara, ASA and EPA included except in the euglena substitution group. For the euglena substitution group, 4 mg *Euglena gracilis* powder (ZhongKe ArnoBio Science & Technology Co., Ltd.) was co‐administered instead of pure EPA. Mice had ad libitum access to DSS‐containing drinking water. Fresh DSS solution was prepared every 2 days to ensure stability. Water intake was monitored daily, and no significant differences in consumption were observed between groups. For dehydration monitoring, mice were monitored daily for signs of dehydration (skin tenting, hunched posture, decreased activity). No severe dehydration requiring euthanasia occurred. All animals survived the 7‐day DSS period. Body weight and disease activity index (DAI) were recorded daily. DAI was calculated as the combined score of weight loss (0‐4), stool consistency (0‐4), and fecal occult blood (0‐4). 24 hours after the end of the DSS period, mice were euthanized, and colon length was measured. Blood and tissue samples were collected for further analysis. No animals were excluded except for technical reasons (poor tissue preservation during histological processing; failure of 16S rDNA amplicon sequencing quality control), with exclusions performed blindly before analysis.

### 2.9 RNA isolation

Total RNA was extracted from fresh animal tissues (10∼50 mg) using the RNA‐easy Isolation Reagent (Cat# R701‐02‐AA, Vazyme, China) following the manufacturer's protocol. RNA purity and integrity were assessed by spectrophotometry and agarose gel electrophoresis. Detailed methods are provided in Supplementary Data 1.

### 2.10 Quantitative real‐time PCR

RT‐qPCR was performed using Taq Pro Universal SYBR qPCR Master Mix (Cat# Q712‐02, Vazyme, China) on a StepOnePlus Real‐Time PCR System (Applied Biosystems, USA). Relative gene expression was calculated using the 2^‐ΔΔCt^ method with *Actb* as the internal control. All the primers are listed in Table S1.

### 2.11 Histological analysis

Tissues were fixed in 4% paraformaldehyde, embedded in paraffin, sectioned at 5 µm, and stained with H&E (Cat# G1076, Servicebio, China) for histopathological evaluation. Colonic sections were also stained with Alcian Blue‐Periodic Acid‐Schiff (AB‐PAS, Cat# G1049‐3, Servicebio, China) for goblet cell and mucus visualization. Histological scoring was performed blindly based on crypt loss, inflammatory infiltration, and mucosal damage (0‐4 for each parameter).

### 2.12 Immunohistochemistry

Colonic sections were deparaffinized, rehydrated, and subjected to antigen retrieval in citrate buffer (pH 6.0) and blocked for endogenous peroxidase with 3% H_2_O_2_. Sections were incubated overnight at 4°C with anti‐ZO‐1 (1:200, Cat# 21773‐1‐AP, Proteintech, China) or anti‐occludin (1:200, Cat# 27260‐1‐AP, Proteintech, China), followed by HRP‐conjugated secondary antibodies. Signals were visualized with DAB and counterstained with hematoxylin.

### 2.13 Biochemical measurements

Serum AST and ALT were measured using commercial kits (Cat# C010‐2 and C009‐1, Nanjing Jiancheng, China) according to the manufacturer's protocols. MPO activity in colon tissues was determined using an MPO assay kit (Cat# A044‐1, Nanjing Jiancheng, China) following the manufacturer's instructions. Spleen mass index was calculated as spleen weight (mg) divided by body weight (g).

### 2.14 16S rDNA amplicon sequencing and microbiota analysis

16S rDNA amplicon sequencing was performed by MetWare (http://www.metware.cn/). Fecal DNA was extracted using a PowerSoil DNA Kit (Qiagen, Germany). The V4 region was amplified with primers 515F and 806R, and libraries were sequenced on an MGI sequencing platform (paired‐end, 2 × 300 bp). Raw reads were processed through QIIME2, with taxonomic assignment against SILVA 138.1. Alpha and beta diversity metrics were calculated using QIIME, and PCoA, NMDS, UPGMA, Adonis and Simper analyses were performed. Detailed methods are provided in Supplementary Data 1.

### 2.15 Statistical analysis

Statistical analyses were performed using GraphPad Prism (version 10.2.0). Data were expressed as mean ± SD. Multiple‐groups comparisons were made using Ordinary one‐way ANOVA multiple comparisons tests, and microbiota data were analyzed using Kruskal‐Wallis with Dunn's post hoc. A *p*‐value < 0.05 was considered statistically significant. Detailed *p*‐values are provided in Table S2.

## 3 RESULTS

### 3.1 Construction and in vitro functional validation of the RvE1 biosynthetic engineered probiotic

RvE1 is generated through a transcellular biosynthetic pathway: EPA is converted to 18*R*‐hydroxyeicosapentaenoic acid (18*R*‐HEPE) by cyclooxygenase 2 (COX2) acetylated by aspirin (acetylsalicylic acid, ASA) in activated endothelial cells; 18*R*‐HEPE is then released into the blood vessel lumen, taken up by neutrophils, and further converted to RvE1 by neutrophil‐expressed 5‐lipoxygenase (5‐LOX).^16^, ^19^ This in vivo biosynthetic process is complex, involving multiple tissue and cell types and their interactions, and is further complicated by the diversity of oxidized PUFAs products and their distinct physiological effects.^18^, ^36^

**FIGURE 1:**
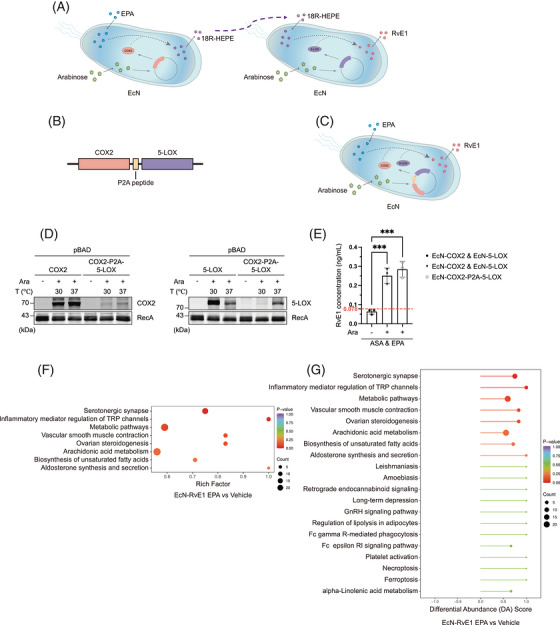
Construction and in vitro functional validation of the RvE1 biosynthetic engineered probiotic. (A) Schematic of EcN‐COX2 and EcN‐5‐LOX construct and the biosynthetic pathway of RvE1 in vitro. (B) Schematic of the structure of pBAD‐COX2‐P2A‐5‐LOX construct. (C) Schematic of EcN‐COX2‐P2A‐5‐LOX construct and the biosynthetic pathway of RvE1 in vitro. (D) Western blot analysis of COX2 and 5‐LOX expression in EcN‐COX2, EcN‐5‐LOX and EcN‐COX2‐P2A‐5‐LOX strains. Bacterial lysates were prepared after induction with 0.4% arabinose for 12 h at the indicated temperatures. (E) Quantification of RvE1 concentration in culture supernatants measured by ELISA. Equal densities of EcN‐COX2 and EcN‐5‐LOX mixed strains or EcN‐COX2‐P2A‐5‐LOX strain were co‐incubated with aspirin (ASA) and the substrate EPA. The red dashed line and number indicate the limit of detection of the ELISA assay. The bars are represented as mean ± SD (*n* = 3 per group). Statistics: Ordinary one‐way ANOVA multiple comparisons tests (ns, not significant, * *p* < .05, ** *p* < .01, *** *p* < .001, **** *p* < .0001). (F) KEGG pathway enrichment analysis of differential oxylipins identified by LC–MS/MS in the EPA treatment group compared with the vehicle control group. The bubble chart shows the Rich Factor for each pathway, with bubble size representing the number of enriched metabolites and colour indicating the adjusted *p*‐value. No pathway reached statistical significance after multiple testing correction (*p* ≥ .05 for all). (G) Differential abundance (DA) score analysis of the enriched KEGG pathways. The DA score reflects the overall directional change of all identified metabolites within each pathway. The length of each horizontal bar represents the absolute DA Score, with bars extending to the right of the central axis indicating net upregulation and bars extending to the left indicating net downregulation. Dot size corresponds to the number of differential metabolites identified in the pathway, and dot colour represents the adjusted *p*‐value.

To achieve efficient and specific prokaryotic biosynthesis of RvE1 using non‐pathogenic *Escherichia coli* Nissle 1917 (EcN) as a non‐invasive oral probiotic delivery platform, we codon‐optimized the key biosynthetic enzyme genes COX2 and 5‐LOX for prokaryotic protein expression (Table S1). We employed the arabinose (Ara)‐inducible pBAD expression system, which enables mild, controllable, and safe induction,^37^ to construct recombinant expression vectors for COX2 and 5‐LOX, which were subsequently introduced into EcN to generate the Ara‐inducible strains EcN‐COX2 and EcN‐5‐LOX (Figure 1A; Figure S1A). Given the complexity and uncertainty associated with the separate induction of the two key enzymes, we further optimized the system by constructing a recombinant pBAD vector in which COX2 and 5‐LOX were tandemly linked via a P2A linker, generating the EcN‐COX2‐P2A‐5‐LOX strain (Figure 1B,C; Figure S1B). Growth curve analysis revealed that the proliferation kinetics of all recombinant strains were essentially indistinguishable from that of wild‐type EcN, indicating that plasmid introduction did not compromise bacterial growth (Figure S1C). To verify inducible expression of the key enzymes, we performed western blot analysis on lysates of bacteria induced with 0.4% Ara for 12 h at different temperatures. Inducible expression of both enzymes was observed in all three strains, with higher expression efficiency at 37°C (Figure 1D).

To determine whether the key enzymes expressed in EcN possessed full catalytic activity for converting EPA to RvE1, we co‐incubated the COX2 acetylating agent ASA and the substrate EPA with either a mixture of Ara‐induced EcN‐COX2 and EcN‐5‐LOX at equal cell densities, or with the Ara‐induced EcN‐COX2‐P2A‐5‐LOX strain. Enzyme‐linked immunosorbent assay (ELISA) of the culture supernatants revealed that both the mixed‐strain system (separate enzyme expression) and the tandem expression strain were capable of catalysing RvE1 synthesis, with the tandem expression strain showing superior efficiency (Figure 1E). Based on these findings, we selected the EcN strain harbouring the COX2‐P2A‐5‐LOX recombinant vector, designated EcN‐RvE1, for further investigation.

To characterize the polyunsaturated fatty acid oxidation metabolism of the EcN‐RvE1 strain, we performed oxylipin analysis by mass spectrometry on the culture supernatants of induced engineered bacteria using EPA as a substrate (Figure S2A,B). Given the challenges associated with oxylipin extraction and identification—including low abundance, structural complexity, numerous isomers and poor stability with susceptibility to oxidation^18^, ^38^, ^39^—we were unable to detect the anticipated product RvE1. Nevertheless, principal component analysis (PCA) revealed clear separation between the EPA‐supplemented group and the solvent control group, indicating differences in oxylipin metabolic profiles (Figure S2C). Despite the absence of statistically significant pathway enrichment (*p* > .05)—probably reflecting the restricted number of detected oxylipins—Kyoto Encyclopedia of Genes and Genomes (KEGG) pathway enrichment analysis of differentially abundant metabolites indicated that metabolic pathways and biosynthesis of unsaturated fatty acids exhibited high enrichment levels. Notably, arachidonic acid metabolism also demonstrated high enrichment, which may be attributable to overlap in pathway‐associated enzymes and metabolites (Figure 1F). Differential abundance score (DA Score) analysis further revealed that all highly enriched metabolic pathways were positively enriched, with an overall upregulation trend in pathway‐associated metabolites (Figure 1G). These results suggested that introduction of the COX2‐P2A‐5‐LOX expression vector remodelled the lipid oxidation metabolic network of the engineered EcN strain.

### 3.2 Validation of in vivo colonization capacity of EcN‐RvE1

**FIGURE 2:**
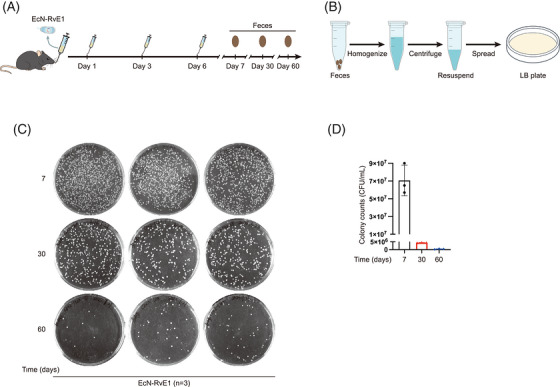
Validation of in vivo colonization capacity of EcN‐RvE1. (A) Schematic representation of the experimental timeline for evaluating intestinal colonization of EcN‐RvE1. (B) Schematic workflow for faecal sample processing and bacterial culture. (C and D) Representative images (C) and quantification (D) of colony growth on agar plates from faecal samples collected at Days 7, 30 and 60 post‐gavage. The bars are represented as mean ± SD (*n* = 3 per group).

In vivo, particularly intestinal residence is a critical determinant for the performance of orally administered probiotic therapeutics. To assess whether EcN‐RvE1 could sustain itself in the gut, we used C57BL/6 mice as a model and administered 10^9^ colony‐forming units (CFUs) of EcN‐RvE1 via intragastric (i.g.) gavage three times within 1 week. Faecal samples were collected at Days 7, 30 and 60 following the final gavage (Figure 2A). Following washing and bacterial isolation, faecal sample suspensions were plated onto antibiotic‐supplemented agar, colonies were enumerated following 24 h of incubation at 37°C with colony‐forming units per millilitre (CFU/mL) quantified (Figure 2B). Colony counts from faecal samples collected at Day 7 reached 7 × 10^7^ CFU/mL, indicating that orally administered EcN‐RvE1 survived normally in the intestine with favourable fitness. Colony formation was also observed on plates from faecal samples collected at Days 30 and 60, with colony counts at Day 60 post‐gavage still reaching 4 × 10^5^ CFU/mL, suggesting that EcN‐RvE1 possessed the capacity for long‐term intestinal persistence (Figure 2C,D).

To confirm that the colonies grown from faecal samples were EcN‐RvE1 itself rather than random colonies arising from antibiotic resistance or horizontal gene transfer of resistance genes, we randomly selected colonies from day 60 samples and performed colony PCR using pBAD vector‐specific primers. Sequencing of the PCR products revealed that the colonies contained both COX2 and 5‐LOX genes, indicating that EcN‐RvE1 was capable of sustained presence in the in vivo environment (Figure S3A).

### 3.3 Therapeutic efficacy of EcN‐RvE1 in LPS‐induced acute inflammation mice

**FIGURE 3:**
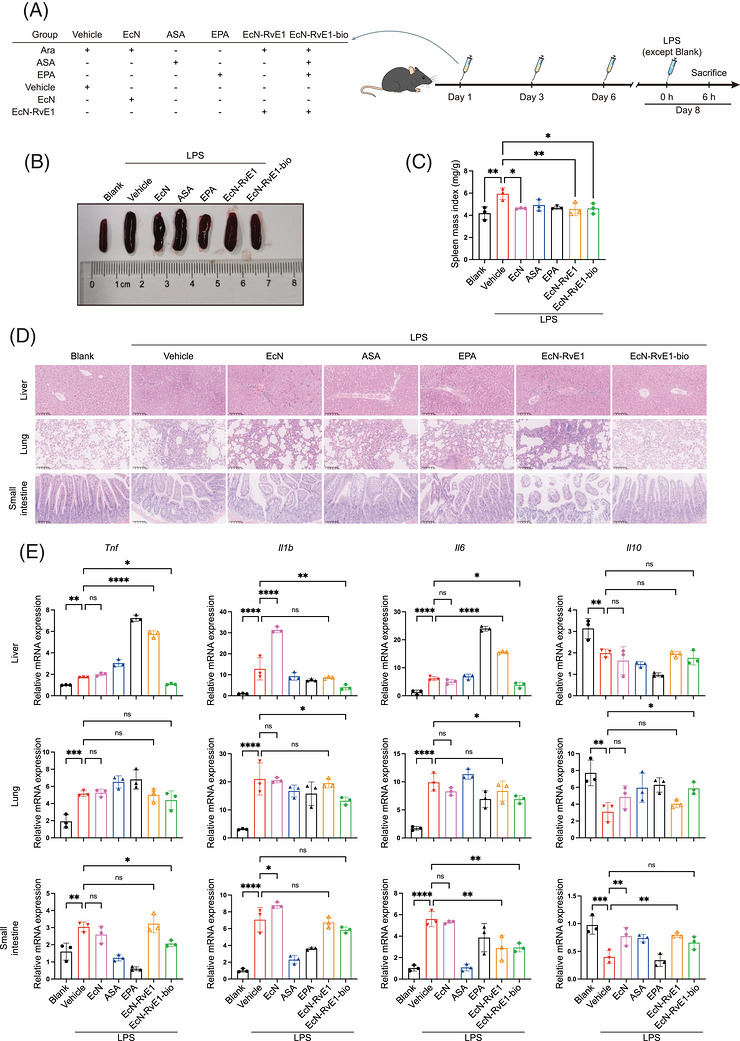
Therapeutic efficacy of EcN‐RvE1 in LPS‐induced acute inflammation mice. (A) Schematic representation of the experimental timeline for the LPS‐induced acute inflammation model. (B and C) Representative images of spleen morphology from each experimental group (B) and corresponding quantification of spleen mass index (spleen weight/body weight ratio, C). The bars are represented as mean ± SD (*n* = 3 per group). Statistics: Ordinary one‐way ANOVA multiple comparisons tests (ns, not significant, * *p* < .05, ** *p* < .01, *** *p* < .001, **** *p* < .0001). (D) Representative H&E staining images of liver, lung and small intestine tissues from each group. The scale bar indicates 100 µm. (E) Relative mRNA expression levels of pro‐inflammatory cytokines (*Tnf*, *Il1b*, *Il6*) and anti‐inflammatory cytokine (*Il10*) in liver, lung and small intestine tissues measured by RT‐qPCR. The bars are represented as mean ± SD (*n* = 3 per group). Statistics: Ordinary one‐way ANOVA multiple comparisons tests (ns, not significant, * *p* < .05, ** *p* < .01, *** *p* < .001, **** *p* < .0001).

To validate the anti‐inflammatory effects of EcN‐RvE1, we first assessed its therapeutic efficacy in a murine model of acute inflammation. The murine model of acute systemic inflammation induced by intraperitoneal or intravenous injection of lipopolysaccharide (LPS) is a well‐established animal model for studying acute inflammatory responses.^40^ Female C57BL/6 mice were assigned to groups that received intragastric (i.g.) gavage three times within 1 week with PBS vehicle, 10^9^ CFUs of EcN, EcN‐RvE1 alone, or EcN‐RvE1 for RvE1 biosynthesis (EcN‐RvE1 + ASA + EPA, designated EcN‐RvE1‐bio). All treatment groups were supplemented with 0.4% arabinose as an inducer. A blank group, an ASA‐only group, and an EPA‐only group were also included as controls. Two days after the final gavage, all mice except the blank controls received an intraperitoneal (i.p.) injection of LPS (5 mg/kg) to elicit acute systemic inflammation, and mice were euthanized for tissue and blood analyses 6 h after injection (Figure 3A). LPS injection resulted in splenic congestion, enlargement and dark red discoloration, with a significantly elevated spleen index compared with the blank group. Notably, the presence of EcN‐RvE1 restored spleen morphology to a state similar to that of the blank group and effectively reversed the increase in spleen index (Figure 3B,C).

We next examined multi‐organ inflammatory responses across groups. H&E staining revealed that LPS stimulation induced hepatic cord disorganization, cellular necrosis and pronounced inflammatory cell infiltration in liver tissue; alveolar congestion, alveolar wall thickening and neutrophil infiltration and aggregation in lung tissue; and structural damage, distorted mucosal villi, intestinal mucosal epithelial swelling, marked separation of the lamina propria with basal layer rupture and necrosis, and lymphocytic and neutrophilic infiltration in the interstitial tissue of the small intestine (Figure 3D). Compared with the blank group, histological scores of small intestinal tissues were markedly increased following LPS stimulation (Figure S4A), and serum aspartate aminotransferase (AST) and alanine aminotransferase (ALT) activities were also significantly elevated (Figure S4B). In contrast, EcN‐RvE1 intervention effectively attenuated tissue damage and inflammatory cell infiltration across all examined tissues, and significantly reduced serum AST and ALT levels (Figure 3D; Figure S4A,B).

Mechanistically, LPS induces systemic inflammation by triggering a burst of pro‐inflammatory mediator expression.^41^ Consistent with this, we observed significantly elevated transcript levels of pro‐inflammatory cytokines such as *Tnf*, *Il1b* and *Il6* in tissue samples from LPS‐treated mice, whereas EcN‐RvE1 intervention robustly suppressed the expression of these cytokines (Figure 3E). Notably, the presence of EcN‐RvE1 also counteracted the LPS‐driven decline in anti‐inflammatory cytokine *Il10* expression, implying that its pro‐resolving action extends beyond the canonical NF‐κB suppression mediated by RvE1‐ChemR23 engagement, and may additionally involve modulation of pathways that sustain anti‐inflammatory cytokine production. Taken together, these results demonstrate that EcN‐RvE1 effectively exerts early pro‑resolving effects at the peak of systemic acute inflammation through inflammation suppression and multi‐organ protection.

### 3.4 Therapeutic efficacy of EcN‐RvE1 in DSS‐induced colitis mice

**FIGURE 4:**
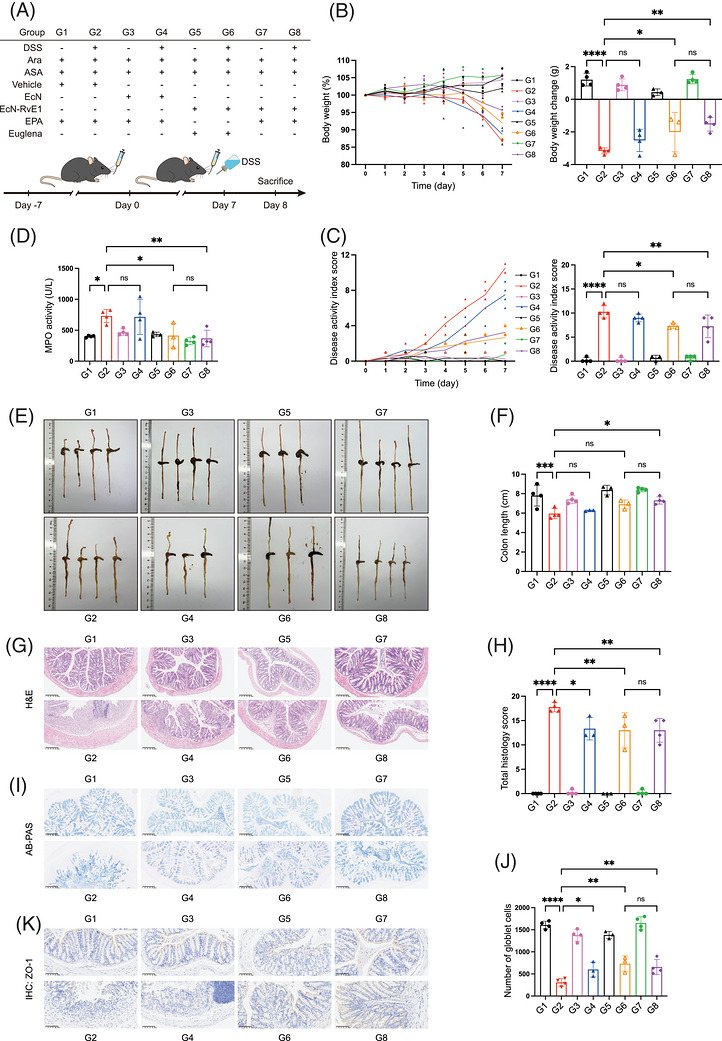
Therapeutic efficacy of EcN‐RvE1 in DSS‐induced colitis mice. (A) Schematic representation of the experimental timeline for the DSS‐induced colitis model. G1, G3, G5 and G7 are non‑DSS controls demonstrating baseline safety and substrate comparability. (B) Body weight changes over the course and day 8 of DSS treatment. Left panel: Data are presented as percentage of initial body weight. Right panel: The bars are represented as mean ± SD (*n* = 3 or 4 per group). Statistics: Ordinary one‐way ANOVA multiple comparisons tests (ns, not significant, * *p* < .05, ** *p* < .01, *** *p* < .001, **** *p* < .0001). (C) Disease activity index (DAI) scores of each experimental group over the course and day 8 of DSS treatment. Right panel: The bars are represented as mean ± SD (*n* = 3 or 4 per group). Statistics: Ordinary one‐way ANOVA multiple comparisons tests (ns, not significant, * *p* < .05, ** *p* < .01, *** *p* < .001, **** *p* < .0001). (D) Myeloperoxidase (MPO) activity of each experimental group measured as a marker of neutrophil infiltration. The bars are represented as mean ± SD (*n* = 3 or 4 per group). Statistics: Ordinary one‐way ANOVA multiple comparisons tests (ns, not significant, * *p* < .05, ** *p* < .01, *** *p* < .001, **** *p* < .0001). Representative images of colon length from each experimental group (E) and corresponding quantification of colon length (F). The bars are represented as mean ± SD (*n* = 3 or 4 per group). Statistics: Ordinary one‐way ANOVA multiple comparisons tests (ns, not significant, * *p* < .05, ** *p* < .01, *** *p* < .001, **** *p* < .0001). (G) Representative H&E staining images of colonic tissue from each group. The scale bar indicates 200 µm. (H) Total histological scores of colonic damage and inflammation from each experimental group. The bars are represented as mean ± SD (*n* = 3 or 4 per group). Statistics: Ordinary one‐way ANOVA multiple comparisons tests (ns, not significant, * *p* < .05, ** *p* < .01, *** *p* < .001, **** *p* < .0001). (I) Representative Alcian Blue‐Periodic Acid‐Schiff (AB‐PAS) staining of colonic sections showing goblet cells and the mucus layer. The scale bar indicates 200 µm. (J) Quantification of goblet cell numbers per crypt. The bars are represented as mean ± SD (*n* = 3 or 4 per group). Statistics: Ordinary one‐way ANOVA multiple comparisons tests (ns, not significant, * *p* < .05, ** *p* < .01, *** *p* < .001, **** *p* < .0001). (K) Representative immunohistochemistry (IHC) images of the tight junction protein ZO‐1 in colonic tissues. The scale bar indicates 100 µm.

We next focussed on elucidating the therapeutic efficacy and intestinal protective effects of EcN‐RvE1 in colitis. To model IBD, we administered 4% dextran sulphate sodium (DSS) in drinking water to female C57BL/6 mice for seven consecutive days, a regimen that closely mimics human ulcerative colitis histopathology.^42^, ^43^ Before and during DSS exposure, mice received three weekly i.g. doses of PBS, EcN, or EcN‐RvE1, with the latter groups receiving Ara, EPA and ASA alongside the bacterial suspension. Mice were euthanized for sample collection 1 day after the final DSS treatment (Figure 4A). To directly confirm the production of RvE1 in vivo, we performed ELISA on colonic tissue homogenates and the results showed significantly elevated RvE1 levels in the presence of EcN‐RvE1 (Figure S5A).

DSS administration caused rapid weight loss, a hallmark of colitis severity. Dynamic monitoring of body weight revealed that EcN‐RvE1 treatment significantly attenuated the extent of DSS‐induced weight loss (Figure 4B). Elevated disease activity index (DAI) represents another characteristic clinical manifestation of DSS treatment, and the DAI score in the EcN‐RvE1 group was significantly lower than that in the model group, indicating a global protective effect against colitis (Figure 4C). We further assessed neutrophil infiltration and intestinal inflammation severity by measuring myeloperoxidase (MPO) activity.^44^ EcN‐RvE1 intervention reversed the DSS‐induced increase in MPO activity, suggesting inhibition of neutrophil infiltration (Figure 4D). EcN‐RvE1 also restored serum AST and ALT levels in colitis mice, although the AST/ALT ratio did not differ significantly, suggesting consistent patterns of colitis‐mediated liver injury with mitigated severity (Figure S5B,C).

DSS‐treated mice exhibited pronounced characteristic intestinal pathologies, including colon length shortening, loss of crypt architecture, reduced goblet cell numbers, diminished mucus production with mucus layer damage and dense inflammatory infiltration of the lamina propria.^43^ To assess the protective impact of EcN‐RvE1 on inflamed colonic tissue, we first analysed morphological features of intestinal samples. Colon length measurements revealed that EcN‐RvE1 intervention ameliorated DSS‐induced colon shortening (Figure 4E,F). Histological evaluation of H&E‐stained sections revealed that DSS caused extensive tissue destruction and inflammatory cell accumulation, reflected in markedly elevated total histological scores relative to the blank group. In contrast, the EcN‐RvE1‐treated group exhibited markedly reduced tissue damage and inflammatory cell infiltration, with significantly decreased total histological scores (Figure 4G,H). Alcian blue‐periodic acid‐Schiff (AB‐PAS) staining of colonic epithelial mucosal layer goblet cell mucin further demonstrated that EcN‐RvE1 ameliorated DSS‐induced mucus layer damage and goblet cell depletion, suggesting a protective effect on colonic mucosal barrier function (Figure 4I,J). At the molecular level, EcN‐RvE1 restored expression of the tight junction proteins zonula occludens‐1 (ZO‐1) and occludin, which are characteristically downregulated following DSS treatment,^45^ further reflecting its role in promoting colonic mucosal healing and barrier function restoration under inflammatory stimulation (Figure 4K; Figure S5D). In addition, EcN‐RvE1 exhibited protective effects against splenic inflammation in DSS‐induced colitis mice, including improved spleen morphology and reversal of the elevated spleen index (Figure S5E,F), consistent with observations in the LPS‐induced acute inflammation model (Figure 3B,C). These results demonstrate that the RvE1 biosynthetic engineered probiotic platform exhibits promising therapeutic efficacy in colonic inflammation and intestinal protection, highlighting its potential for clinical translation.

Notably, the source of precursor material represents another challenge for the clinical application of pro‐resolving mediators. Traditional sources of omega‐3 polyunsaturated fatty acids rely primarily on deep‐sea fish oil, which faces limitations in supply capacity and sustainability.^46^ In contrast, directly synthesized and omega‐3‐rich photosynthetic protist, such as *Euglena gracilis* (photosynthetic protist with eyespot), represent a cleaner and more sustainable alternative.^47^, ^48^ Furthermore, the β‐glucan uniquely present in *Euglena gracilis* possesses potent immunomodulatory properties that enhance host resistance.^49^ To determine whether EcN‐RvE1 using photosynthetic protist‐derived omega‐3 as a substrate similarly exhibits protective effects against DSS‐induced colitis, we included a group in which EPA was replaced with euglena powder (Figure 4A) and the elevated RvE1 level in the gut microenvironment can also be detected (Figure S5A). Euglena treatment demonstrated comparable efficacy to EPA in attenuating body weight loss and DAI elevation (Figure 4B,C), with similar effects observed on MPO activity and AST/ALT levels (Figure 4D; Figure S5B,C). The euglena substitution group also showed comparable efficacy to the EPA‐treated group with respect to inflammation resolution, restoration of colonic barrier function, and protective effects on intestinal and splenic tissues (Figure 4E–K; Figure S5D–F), highlighting the capacity of the EcN‐RvE1 platform combined with photosynthetic protist‐derived omega‐3 substrates to effectively inhibit inflammatory injury.

### 3.5 EcN‐RvE1 correlates with the amelioration of gut microbiota dysbiosis in DSS‐induced colitis mice

The gut microbiota plays a critical role in maintaining intestinal homeostasis, regulating immune function, and balancing inflammation initiation and resolution.^50^ Having established that the RvE1 biosynthetic engineered strain using either EPA or euglena as a substrate significantly ameliorated DSS‐induced colitis, we next investigated whether EcN‐RvE1 modulated and improved the dysbiotic gut microbiota in colitis mice. To characterize the gut microbial composition and community structure, we performed 16S rDNA amplicon sequencing on faecal samples from DSS‐challenged mice^51^, ^52^, ^53^ (Figure 4A). Rarefaction curves reached saturation beyond 30 000 reads, and Good's coverage values exceeded 0.99 across all samples, confirming sufficient sequencing depth (Figure S6A; Table S2). Rank abundance curves revealed species distribution patterns consistent with typical gut microbiota characteristics (Figure S6B).

**FIGURE 5:**
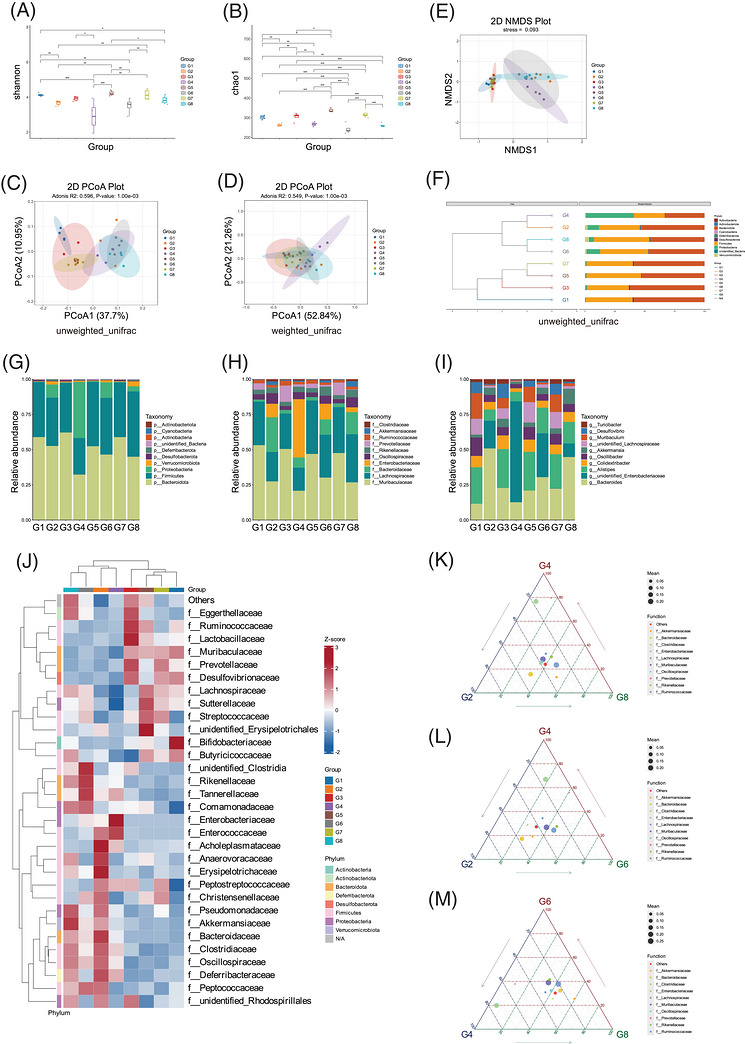
EcN‐RvE1 correlates with the amelioration of gut microbiota dysbiosis in DSS‐induced colitis mice. (A and B) Alpha diversity analysis shown as Shannon index (A) and Chao1 index (B) across experimental groups. The Shannon index reflects both species richness and evenness, and the Chao1 index estimates species richness based on the number of rare species (singletons and doubletons). Data are presented as box plots, with the horizontal line within each box indicating the median value (*n* = 3 or 4 per group). Statistics: Kruskal–Wallis rank test (* *p* < .05, ** *p* < .01, *** *p* < .001). (C and D) Principal Co‐ordinates Analysis (PCoA) of gut microbiota based on unweighted UniFrac distances (C) and weighted UniFrac distances (D). The percentage of variance explained by each principal co‐ordinate, and Adonis analysis are indicated. (E) Non‐metric multi‐dimensional scaling (NMDS) analysis of gut microbiota composition. A stress value below 0.2 indicates that the NMDS plot accurately reflects the differences between samples. (F) Unweighted pair‐group method with arithmetic mean (UPGMA) hierarchical clustering tree based on unweighted UniFrac distances, integrated with phylum‐level relative abundance distributions. The clustering tree shows overall structural relatedness among samples from different groups. (G–I) Relative abundance of bacterial taxa at the phylum (G), family (H) and genus (I) level across experimental groups. Top 10 taxonomy for maximum abundance shown in the legend. (J) Heatmap showing the distribution of differentially abundant species at the family level across experimental groups. The colour gradient represents Z‐score standardized relative abundance, with red indicating higher abundance and blue indicating lower abundance. Hierarchical clustering of both samples and species is shown. (K–M) Ternary plot analysis at the family level comparing the distribution of dominant species among three indicated groups. Each vertex represents a group, each circle represents a bacterial family, and circle size is proportional to relative abundance. Circles positioned closer to a vertex indicate higher abundance in that group.

Diversity analyses showed that EcN‐RvE1 treatment, using either EPA or euglena as a substrate, ameliorated DSS‐induced impairment of gut microbiota diversity, suggesting a protective effect on microbial diversity under inflammatory conditions (Figure 5A,B; Figure S6C,D). Principal coordinates analysis (PCoA) revealed more pronounced intergroup separation based on unweighted UniFrac distances (Figure 5C, *R* ^2^ = .596, *p* = .001) compared with weighted UniFrac distances (Figure 5D, *R* ^2^ = .549, *p *= .001), suggesting that changes in rare species composition were the primary drivers of microbial community structure alterations, with DSS treatment leading to significant microbial dysbiosis. Non‐metric multidimensional scaling (NMDS) analysis further corroborated these findings (Figure 5E, stress = .093). Distance matrix heatmaps illustrating species similarity patterns among groups were consistent with PCoA findings (Figure S6E–H). Unweighted pair‐group method with arithmetic mean (UPGMA) hierarchical clustering showed that, although DSS‐treated groups formed a distinct branch, EcN‐RvE1 intervention still showed a trend toward improving overall gut microbiota structure (Figure 5F; Figure S6I). Notably, when integrated with species relative abundance at the phylum level, UPGMA analysis demonstrated that the presence of EcN‐RvE1 shifted DSS‐mediated microbial alterations toward the control group, suggesting that the protective effects of the engineered probiotic treatment were closely associated with changes in gut microbiota structure.

To further elucidate the potential mechanisms by which EcN‐RvE1 exerted its anti‐inflammatory effects through amelioration of gut microbiota dysbiosis, we analysed specific microbial species composition. Species annotation revealed detailed taxonomic composition at the amplicon sequence variant (ASV) level for each sample (Figure S7A,B; Table S2). At the phylum level, DSS treatment markedly depleted the relative abundance of *Firmicutes* and expanded *Proteobacteria*, consistent with previously reported microbial characteristics of DSS‐induced colitis models.^54^ EcN‐RvE1 intervention significantly restored *Firmicutes* levels and suppressed *Proteobacteria* overgrowth (Figure 5G). Family (Figure 5H) and genus (Figure 5I) levels analyses further revealed that DSS promoted the expansion of the pathogenic family *Enterobacteriaceae*, whereas EcN‐RvE1 enriched beneficial taxa, including *Lachnospiraceae*, *Akkermansia* and *Alistipes*. Heatmap analysis further refined the distribution patterns of differentially abundant species among groups, revealing alterations induced by EcN‐RvE1 intervention and suggesting that it associated with the amelioration of DSS‐induced dysbiosis by restoring core beneficial bacterial abundance (Figure 5J; Figure S8A). Consistently, similarity percentage (Simper) analysis quantifying the major species contributing to intergroup separation identified *Lachnospiraceae* and *Akkermansia* as key contributors driving the separation between the engineered probiotic intervention group and the DSS group (Figure S8B–G).

To evaluate substrate‐specific effects, we compared ternary plots of the top 10 taxa at family and genus levels for EPA versus euglena supplementation. Both substrates effectively associated with the amelioration of DSS‐induced dysbiosis, with EPA treatment exhibiting more precise microbiota‐modulating capacity through specific enrichment of key butyrate‐producing beneficial bacteria, further substantiating the superior efficacy of EcN‐RvE1 in restoring microbial homeostasis (Figure 5K–M; Figure S8H–J). Collectively, these results reveal the potential mechanisms by which the RvE1 biosynthetic engineered probiotic correlates with the amelioration of gut microbiota dysbiosis in colitis mice—through restoration of microbial diversity, promotion of colonization by core butyrate‐producing bacteria and intestinal barrier‐protecting bacteria, and suppression of pro‐inflammatory bacterial expansion—thereby exerting effective anti‐inflammatory therapeutic effects.

## 4 DISCUSSION

The treatment and management of inflammatory diseases have long been constrained by the systemic side effects of conventional anti‐inflammatory drugs and their inability to reverse established tissue damage. The discovery of SPMs has introduced a paradigm shift in inflammation therapy, transitioning from direct suppression of inflammation to active promotion of its resolution. As a potent member of the SPMs family derived from the omega‐3 polyunsaturated fatty acid EPA, RvE1 has demonstrated therapeutic potential in various inflammatory disease models. However, its clinical translation is hindered by pharmacokinetic limitations due to chemical instability and high production costs associated with complex total synthesis. The integration of synthetic biology with engineered probiotic platforms offers a safe, effective and cost‐effective alternative. Using the non‐pathogenic EcN as a chassis probiotic, we developed a biosynthetic engineered probiotic platform, EcN‐RvE1, that circumvents the delivery bottlenecks of exogenous RvE1 by enabling sustained in situ biosynthesis. We validated its systemic protective effects in an acute inflammation murine model and its intestinal protective effects in a colitis murine model. Importantly, long‐term intestinal persistence and non‐invasive oral administration align closely with the disease management needs of both acute inflammation and chronic inflammatory conditions. Furthermore, leveraging the inherent safety advantages of probiotics, this strategy not only addresses the application bottlenecks of RvE1 but also establishes a versatile framework for translating SPMs‑based therapies.

The therapeutic efficacy of EcN‐RvE1 arises from the synergistic action of multiple mechanisms. Notably, EcN‐RvE1 lowered the transcript levels of the pro‐inflammatory cytokines *Tnf*, *Il1b* and *Il6* (Figure 3E), consistent with the established mechanism by which RvE1 inhibits the NF‐κB pathway through the ChemR23 receptor.^19^, ^20^ Concurrently, EcN‐RvE1 treatment associated with the amelioration of gut microbiota dysbiosis in colitis mice, as evidenced by restored diversity, reduced abundance of pathogenic bacteria, and enrichment of beneficial bacteria (Figure 5). This suggests that the anti‐inflammatory effects of RvE1 extend beyond direct modulation of immune cells to include indirect effects through remodelling of the microbiota–immune axis. The gut microbiome is increasingly viewed as a key regulator of systemic immunity, exerting influence through barrier maintenance, metabolite production and crosstalk with innate and adaptive immune cells. Prior work has demonstrated that probiotics and their engineered counterparts can attenuate systemic inflammation by reshaping the gut microbiota, tightening epithelial barriers and lowering circulating endotoxin levels.^55^, ^56^, ^57^ RvE1 itself has been reported to bolster intestinal barrier function and to upregulate intestinal alkaline phosphatase, which detoxifies LPS and may indirectly reduce systemic endotoxemia.^58^ In our study, EcN‐RvE1 treatment significantly restored gut microbiota composition, including enrichment of butyrate‐producing *Lachnospiraceae* and barrier‐enhancing *Akkermansia* (Figure 5). These organisms generate metabolites—such as short‑chain fatty acids—that can reach the circulation and influence systemic immune status. Hence, EcN‐RvE1 may act through a dual mechanism: locally via RvE1 absorption, and indirectly via microbiota‐mediated systemic immunomodulation. Nonetheless, whether RvE1 directly modulates microbial composition or whether the observed changes are secondary to reduced inflammation remains to be determined. Future studies employing faecal microbiota transplantation (FMT) or in vitro culture of gut bacteria with EcN‐RvE1 will address causality.

The combination of euglena, a microalga‐derived source of EPA, with EcN‐RvE1 demonstrated the potential of a synbiotic formulation. Notably, we showed for the first time that co‐administration of euglena powder also enabled the protective effects of EcN‐RvE1 (Figure 4). This finding carries important translational implications. On the one hand, as a microalga‐derived omega‐3 source, *Euglena gracilis* offers advantages in accessibility and sustainability compared with traditional deep‐sea fish oil‐derived omega‐3 sources. Moreover, the β‐glucan uniquely present in *Euglena gracilis* possesses potent immunomodulatory properties that enhance host resistance. On the other hand, the combination of a probiotic (EcN‐RvE1) with a prebiotic/substrate (*Euglena gracilis*) constitutes a genuine synbiotic system, opening new avenues for nutritional intervention strategies in inflammatory diseases.

Several limitations of this study warrant consideration. Post‑hoc power analysis indicated very large effect sizes for primary endpoints. Nevertheless, the statistical power for some comparisons was below the conventional threshold due to small sample sizes (*n* = 3–4 per group). We have therefore committed to using larger cohorts (*n* ≥ 6) with unified sample sizes for all endpoints in future validation studies. A ‘no arabinose’ control was not included because the pBAD promoter exhibits basal leaky expression.^37^ Even without arabinose, trace RvE1 might be produced. Importantly, the EcN‑only control showed no protective effect, confirming that the therapeutic benefit is specifically dependent on the introduced RvE1 biosynthesis pathway rather than the EcN chassis itself. As for the LPS model, while our data show reduced inflammation at 6 h, active resolution mechanisms (e.g., neutrophil clearance, efferocytosis) were not assessed. We have therefore described the effects as anti‐inflammatory with early pro‐resolving potential. Future studies should examine later time points to fully characterize resolution kinetics. Additionally, a RvE1‑deficient mutant would provide definitive specificity evidence. A loss‐of‐function control with inactive COX2 or 5‐LOX mutant need to be generated for future investigations.

Although the key enzyme expression system in EcN‐RvE1 uses the well‐controlled arabinose‐inducible promoter, its expression stability and duration in vivo warrant systematic optimization. Furthermore, the current engineered strain architecture involves co‐expression of two key enzymes to achieve catalytic function, with COX2 additionally requiring aspirin supplementation for acetylation, resulting in a complex system. Future optimization of this platform should consider streamlining the enzymatic catalytic system through structural biology approaches to achieve more sustained RvE1 production while improving patient compliance. As discussed above, engineering aspirin‑independent COX2 variants and EPA‑producing probiotics could further enhance clinical translatability. In addition, future pharmacokinetic studies measuring aspirin and RvE1 levels in the gut lumen and portal circulation using more sensitive LC‑MS/MS will refine dosing and provide direct evidence of absorption. Standard rodent chow contains ∼0.5%–1% omega‐3 PUFAs, which may provide basal substrate. However, to achieve therapeutic levels, we supplemented with EPA or Euglena. Future engineering of an EPA‐producing probiotic could eliminate this requirement. Meanwhile, the use of an ampicillin resistance gene is common in preclinical proof‑of‑concept studies but is not acceptable for clinical translation. Our future iterations will integrate the expression cassette into the chromosome or employ a food‑grade selection system. Potential influences of sex and aging on the efficacy of the engineered probiotic should also be acknowledged as limitations of this study, as independent sex‐ and age‐specific analyses were not conducted in the inflammatory murine models. Additionally, the long‐term efficacy and safety of EcN‐RvE1 in chronic inflammatory settings, along with its expanded application to other inflammatory diseases such as rheumatoid arthritis and neuroinflammation, warrant further investigation, while the direct visualization using bioluminescent or fluorescent reporters would provide more definitive evidence of persistence and spatial distribution.

In summary, we reported an RvE1 biosynthetic engineered probiotic platform, EcN‐RvE1, based on the probiotic EcN. We demonstrated that it alleviated acute inflammation and colitis through dual mechanisms involving modulation of inflammatory cytokines and amelioration of gut microbiota dysbiosis, and that it could synergize with the photosynthetic protist‐derived PUFAs source euglena to form a potential synbiotic formulation. This platform exhibited promise as an oral therapeutic agent that promoted inflammation resolution and tissue protection, offering a feasible strategy to overcome the application bottlenecks of SPMs‐based therapeutics and opening new directions for precision intervention in inflammatory diseases.

## AUTHOR CONTRIBUTIONS

Xiaoxiao Li, Jiejing Lin, Jin Li and Zhonghan Yang conceived the ideas and designed the experiments. Xiaoxiao Li, Jiejing Lin, Jin Li and Zhonghan Yang wrote the paper. Xiaoxiao Li, Jiejing Lin, Qingqing Liu, Qingqing Liu, Qian Wu, Zewei Zhao, Yi Cai, Shengliang Lin, Zijie Zheng and Xinpan Chen performed experiments or performed data analysis. All authors read and approved the final manuscript.

## CONFLICT OF INTEREST STATEMENT

The authors declare no conflicts of interests.

## ETHICS STATEMENT

All female C57BL/6 mice were cared and performed according to the instructions and approval of the Institutional Animal Care and Use Committee of Sun Yat‐sen University (approval number: SYSU‐IACUC‐MED‐2023‐B113).

## Supporting information



Supporting Information

**Figure S1**: Construction and characterization of recombinant expression vectors and engineered strains, related to Figure 1. (A and B) Plasmid profile of the recombinant arabinose‐inducible araBAD promoter containing pBAD expression vector for COX2 and 5‐LOX (A) or COX2‐P2A‐5‐LOX (B). (C) Growth curves of wild‐type EcN and recombinant strains. Left panel: Comparison of EcN, EcN‐COX2 and EcN‐5‐LOX. Right panel: Comparison of EcN and EcN‐COX2‐P2A‐5‐LOX. Bacterial growth was monitored by measuring optical density at 600 nm (OD_600_) over 36 h.

**Figure S2**: Oxylipin analysis of EcN‐RvE1 culture supernatants by LC‐MS/MS, related to Figure 1. (A and B) Western blot analysis confirming the expression of COX2 and 5‐LOX (A) and RvE1 concentration in culture supernatants measured by ELISA (B) in EcN‐RvE1 sample for oxylipin analysis induced with arabinose (Ara) and ASA. The red dashed line and number indicate the limit of detection of the ELISA assay. The bars are represented as mean ± SD (*n* = 3 per group). (C) Principal component analysis (PCA) of oxylipin profiles detected by LC‐MS/MS. PC1 (first principal component) and PC2 (second principal component) are shown on the *x*‐ and *y*‐axes, respectively, with the percentages indicating the proportion of variance explained by each principal component (70.32% for PC1 and 17.79% for PC2). Each dot represents an individual sample, with samples from the same group coloured identically.

**Figure S3**: Molecular confirmation of EcN‐RvE1 colonization by colony PCR, related to Figure 2. (A) Representative sequencing chromatogram of colony PCR products from randomly selected colonies grown from faecal samples collected at day 60 post‐gavage. PCR was performed using gene‐specific primers for COX2 and 5‐LOX.

**Figure S4**: Total histology score and serum liver enzyme activity levels in LPS‐induced acute inflammation model, related to Figure 3. (A) Total histological scores of small intestine tissues from each experimental group. Tissue sections were scored based on parameters including structural damage, inflammatory cell infiltration and mucosal integrity. The bars are represented as mean ± SD (*n* = 3 per group). Statistics: Ordinary one‐way ANOVA multiple comparisons tests (ns, not significant, * *p* < .05, ** *p* < .01, *** *p* < .001, **** *p* < .0001). (B) Serum activity levels of aspartate aminotransferase (AST) and alanine aminotransferase (ALT) measured in each group. The bars are represented as mean ± SD (*n* = 3 per group). Statistics: Ordinary one‐way ANOVA multiple comparisons tests (ns, not significant, * *p* < .05, ** *p* < .01, *** *p* < .001, **** *p* < .0001).

**Figure S5**: Direct detection of RvE1 in colonic tissues, serum liver enzyme levels, tight junction protein expression, and spleen index in DSS‐induced colitis model, related to Figure 4. (A) RvE1 concentration in colonic tissue homogenates measured by ELISA in indicated experimental group. The bars are represented as mean ± SD (*n* = 3 or 4 per group). Statistics: Ordinary one‐way ANOVA multiple comparisons tests (ns, not significant, * *p* < .05, ** *p* < .01, *** *p* < .001, **** *p* < .0001). (B and C) Serum activity levels of AST and ALT (B) and AST/ALT ratio (C) measured in each experimental group. The bars are represented as mean ± SD (*n* = 3 or 4 per group). Statistics: Ordinary one‐way ANOVA multiple comparisons tests (ns, not significant, * *p* < .05, ** *p* < .01, *** *p* < .001, **** *p* < 0.0001). (D) Representative IHC images of the tight junction protein Occludin in colonic tissues from indicated groups. The scale bar indicates 100 µm. (E and F) Representative images of spleen morphology from each experimental group (E) and corresponding quantification of spleen mass index (F). The bars are represented as mean ± SD (*n* = 3 or 4 per group). Statistics: Ordinary one‐way ANOVA multiple comparisons tests (ns, not significant, * *p* < .05, ** *p* < .01, *** *p* < .001, **** *p* < .0001).

**Figure S6**: Rarefaction curves, rank abundance and beta diversity analyses of gut microbiota in DSS‐induced colitis model, related to Figure 5. (A) Rarefaction curves of 16S rDNA amplicon sequencing data for all group samples. The curves show the number of ASVs (Amplicon Sequence Variants) as a function of sequencing reads. All curves plateaued after approximately 30 000 reads, indicating that sequencing depth was sufficient to capture the majority of microbial diversity. (B) Rank abundance curves showing species richness and evenness across experimental groups. The width of the curve reflects species richness, and the smoothness reflects species evenness. (C and D) Alpha diversity analysis shown as observed ASV count (C) and PD whole tree (phylogenetic diversity) index (D) across experimental groups. The observed ASV count directly reflects species richness, and the PD whole tree index measures the sum of branch lengths in the phylogenetic tree, reflecting the evolutionary diversity of the microbial community. Data are presented as box plots, with the horizontal line within each box indicating the median value (*n* = 3 or 4 per group). Statistics: Kruskal–Wallis rank test (* *p* < .05, ** *p* < .01, *** *p* < .001). (E–H) Distance matrix heatmap of beta diversity based on weighted UniFrac distances (E), unweighted UniFrac distances (F), Bray–Curtis distances (G) and binary Jaccard distances (H). In the upper triangle, circles represent pairwise beta diversity between groups, with smaller and redder circles indicating smaller distances and thus lower compositional dissimilarity. The lower triangle uses colour intensity and circle area to convey the same information. (I) UPGMA hierarchical clustering tree based on weighted UniFrac distances, integrated with phylum‐level relative abundance distributions. The clustering tree shows overall structural relatedness among samples from different groups.

**Figure S7**: ASV distribution and species annotation statistics, related to Figure 5. (A) Venn diagram showing the distribution of unique and shared ASVs among selected groups. Each circle represents a group, and overlapping regions indicate the number of ASVs shared between groups. The numbers in non‐overlapping regions represent group‐specific ASVs. (B) Species annotation statistics showing the number of taxa annotated at each taxonomic level (Phylum, Class, Order, Family, Genus, Species) for each sample group. The bar chart displays the count of identified taxa per group across classification levels, reflecting the depth of taxonomic annotation achieved.

**Figure S8**: Differential abundance and similarity percentage analysis of gut microbiota, related to Figure 5. (A) Heatmap showing the distribution of differentially abundant species at the genus level across experimental groups. The colour gradient represents Z‐score standardized relative abundance, with red indicating higher abundance and blue indicating lower abundance. Hierarchical clustering of both samples and genera is shown. (B–G) Similarity percentage (Simper) analysis showing the contribution of bacterial families (B–D) or genera (E–G) to the dissimilarity between G1 and G2 (B and E), G2 and G8 (C and F) and G2 and G6 (D and G). The bar chart displays the top contributing families or genera. (H–J) Ternary plot analysis at the genus level comparing the distribution of dominant species among three indicated groups. Each vertex represents a group, each circle represents a bacterial genus, and circle size is proportional to relative abundance. Circles positioned closer to a vertex indicate higher abundance in that group.

Supporting Information

Supporting Information

Supporting Information

## Data Availability

All data generated or analysed during this study are included in this published article. All other data are available from the corresponding author upon reasonable request.
