# Supplementary material for: Engineered probiotics platform for resolvin E1 biosynthesis confers protection against inflammatory disease
Source: Clin Transl Med. 2026 Jul 19;16(7):e70746. doi: 10.1002/ctm2.70746 (PMC13382362; doi:10.1002/ctm2.70746)
Supplement: Supplementary file 1 — Supporting Information [file CTM2-16-e70746-s005.pdf]

Engineered probiotics platform for resolvin E1 biosynthesis confers protection against inflammatory disease

Supplementary Data 1

Materials and methods: Detailed methods

RvE1 biosynthesis and quantification in vitro

RvE1 concentration was quantified using a commercially available enzyme-linked immunosorbent assay (ELISA) kit (Cat# EK11191, Signalway Antibody, USA) according to the manufacturer's instructions. Briefly, 100 μL samples or standards (0-10 ng/mL) were added to pre-coated 96-well microplates and incubated at 37°C for 2 h. After washing three times with Wash Buffer (250 μL per well), 100 μL of biotin-conjugated anti-RvE1 antibody (1×) was added to each well and incubated at 37°C for 1 h. Following three washes, 100 μL of streptavidin-horseradish peroxidase (HRP) conjugate (1×) was added and incubated at 37°C for 1 h. The plate was washed five times, and 100 μL of substrate solution was added to each well. After incubation at 37°C for 15-20 min in the dark, the reaction was terminated by adding 50 μL of stop solution. The optical density was measured at 450 nm with wavelength correction at 570 nm using a microplate reader within 5 min. The limit of detection was 0.078 ng/mL. All samples were assayed in duplicate. A four-parameter logistic (4-PL) standard curve was generated using Curve Expert software. RvE1 concentrations were calculated by interpolating sample OD values against the standard curve. This assay has high sensitivity and excellent specificity for detection of Human RvE1. No significant cross-reactivity or interference between Human RvE1 and analogues was observed.

Oxylipin analysis by mass spectrometry

Culture supernatants from EcN-RvE1 induced with 0.4% arabinose, ASA, and EPA (or vehicle control) were subjected to oxylipin profiling. Eicosanoids contents were detected by MetWare (http://www.metware.cn/) based on the AB Sciex QTRAP 6500

LC-MS/MS platform.

Chemicals and reagents:

All eicosanoids and deuterated internal standards were purchased from Cayman Chemical. HPLC-grade acetonitrile (ACN) and methanol (MeOH) were purchased from Merck (Darmstadt, Germany). MilliQ water (Millipore, Bradford, USA) was used in all experiments. Acetic acid was purchased from Sigma-Aldrich. CNW Poly-Sery MAX SPE cartridges were from ANPEL Co. (Shanghai, PRC). The stock solutions of standards were prepared at the concentration of 5 μg/mL in MeOH. All stock solutions were stored at -80°C. The stock solutions were diluted with MeOH to working solutions before analysis.

Sample preparation and extraction:

Aliquots of 300 μL aquaculture water sample, add 200 μL internal standard extractant, and shake at 2,500 r/min for 5 min at room temperature. Centrifuge at 12,000 r/min for 10 min at 4°C, and transfer the supernatant. Add 100 μL of the internal standard extractant to the remaining residue, vortex for 5 minutes, and centrifuge at 12,000 r/min for 5 minutes at 4°C, then combine the supernatant. The eicosanoids in supernatants were extracted using Poly-Sery MAX SPEcolumns (ANPEL). Prior to analysis, the eluent was dried under vacuum and redissolvedin 100 μL of methanol/water (1:1, v/v) for UPLC/MS/MS analysis.

HPLC Conditions:

The sample extracts were analyzed using an LC-ESI-MS/MS system (UPLC, ExionLC AD, https://sciex.com.cn/; MS, QTRAP® 6500+ System, https://sciex.com/). The analytical conditions were as follows, HPLC: column, Waters ACQUITY UPLC HSS T3 C18 (100 mm×2.1 mm i.d., 1.8 μm); solvent system, water with 0.04% acetic acid (A), acetonitrile with 0.04% acetic acid (B); The gradient was 0-2.0 min from 0.1% to 30% B; 2.0-4.0 min to 50% B; 4.0-5.5 min to 99% B, which was maintained for 1.5 min; and 6.0-7.0 min reduced to 0.1% B and maintained for 3.0 min. flow rate, 0.4 mL/min; temperature, 40°C; injection volume: 10 μL.

ESI-MS/MS Conditions:

Linear ion trap (LIT) and triple quadrupole (QQQ) scans were acquired on a triple

quadrupole-linear ion trap mass spectrometer (QTRAP), QTRAP® 6500+ LC-MS/MS System, equipped with an ESI Turbo Ion-Spray interface, operating in negative ion mode and controlled by Analyst 1.6.3 software (Sciex). The ESI source operation parameters were as follows: ion source, ESI-; source temperature 550°C; ion spray voltage (IS) -4,500 V; curtain gas (CUR) was set at 35 psi, respectively. Eicosanoids were analyzed using scheduled multiple reaction monitoring (MRM). Data acquisitions were performed using Analyst 1.6.3 software (Sciex). Multiquant 3.0.3 software (Sciex) was used to quantify all metabolites. Mass spectrometer parameters including the declustering potentials (DP) and collision energies (CE) for individual MRM transitions were done with further DP and CE optimization. A specific set of MRM transitions were monitored for each period according to the metabolites eluted within this period.

MS Data Processing:

Unsupervised PCA (principal component analysis) was performed by statistics function prcomp within R (www.r-project.org). The data was unit variance scaled before unsupervised PCA. Significantly regulated metabolites between groups were determined by VIP and absolute Log_2FC (fold change). VIP values were extracted from OPLS-DA result, which also contain score plots and permutation plots, was generated using R package MetaboAnalystR. The data was mean centering before OPLS-DA. In order to avoid overfitting, a permutation test (200 permutations) was performed. Identified metabolites were annotated using KEGG compound database (http://www.kegg.jp/kegg/ compound/), annotated metabolites were then mapped to KEGG Pathway database (http://www.kegg.jp/kegg/pathway.html). Pathways with significantly regulated metabolites mapped to were then fed into MSEA (metabolite sets enrichment analysis), their significance was determined by hypergeometric test's P-Values.

RNA isolation

Total RNA was extracted from fresh animal tissues (10~50 mg) using the RNA-easy Isolation Reagent (Cat# R701-02-AA, Vazyme, China) following the manufacturer's

protocol. Briefly, the tissues were snap-frozen in liquid nitrogen and ground into a fine powder in a liquid nitrogen-precooled mortar. The powder was transferred to an RNase-free centrifuge tube, and 500 μL of RNA-easy Isolation Reagent was added per 25 mg of tissue for complete lysis by vigorous vortexing. RNase-free ddH_2O (2/5 volume of the reagent) was added to the lysate, mixed thoroughly, and incubated at room temperature for 5 min. The mixture was centrifuged at 12,000 × g for 15 min at room temperature, and the upper aqueous phase containing RNA was carefully transferred to a new RNase-free centrifuge tube. An equal volume of isopropanol was added to the aqueous phase, mixed well, and incubated at room temperature for 10 min, followed by centrifugation at 12,000 × g for 10 min at room temperature. The resulting RNA precipitate was rinsed twice with 500 μL of 75% RNase-free ethanol (centrifuged at 8,000 × g for 3 min at room temperature after each rinse). After complete removal of the ethanol supernatant, the precipitate was air-dried at room temperature for 2~3 min (to avoid over-drying) and dissolved in an appropriate volume of RNase-free ddH_2O by vortexing for 3 min at room temperature. The purity and concentration of RNA were determined using a spectrophotometer. RNA integrity was verified by 1% agarose gel electrophoresis. All operations were performed in an RNase-free environment, and the extracted RNA was aliquoted and stored at -80°C for subsequent experiments.

16S rDNA amplicon sequencing and microbiota analysis

Fecal samples collected from DSS colitis experiment mice were subjected to 16S rDNA amplicon sequencing detected by MetWare (http://www.metware.cn/). In brief, fecal DNA was extracted using a PowerSoil DNA Kit (Qiagen, Germany) following the manufacturer's instructions. The V4 region of the 16S rRNA gene was amplified using primers 515F (5′-GTGCCAGCMGCCGCGGTAA-3′) and 806R (5′-GGACTACHVGGGTWTCTAAT-3′). PCR products were purified and libraries were constructed using the VAHTS® Universal Plus DNA Library Prep Kit for MGI V2 (Novizan, China). Sequencing was performed on an MGI sequencing platform (paired-end, 2 × 300 bp). Raw reads were demultiplexed and quality-filtered using

fastp, followed by paired-end assembly using FLASH. Chimeric sequences were detected and removed using UCHIME against the SILVA database. Amplicon sequence variants (ASVs) were generated using the Deblur algorithm within the QIIME2 pipeline. Taxonomic assignment was performed using the Mothur algorithm against the SILVA 138.1 database. Alpha diversity indices (observed ASVs, Shannon, Chao1, PD_whole_tree) and beta diversity metrics (unweighted UniFrac, weighted UniFrac, Bray-Curtis, binary Jaccard) were calculated using QIIME. Principal coordinates analysis (PCoA), non-metric multidimensional scaling (NMDS), and UPGMA hierarchical clustering were performed based on distance matrices. Adonis (PERMANOVA) was used to test the significance of group differences. Similarity percentage (Simper) analysis was conducted to identify taxa contributing most to intergroup dissimilarity. Ternary plots were generated using the vcd package in R. Detailed methods are provided in Supplementary Data 1.

Methods

Contents

1 Sequencing · **1**

1.1 Illumina sequencing 1

1.2 MGI sequencing 3

2 Data analysis · **4**

2.1 Paired-end reads assembly and quality control 4

2.2 OTU/ASV and Species annotation 5

3 Alpha Diversity · **6**

4 Beta Diversity · **7**

Reference · **7**

1 Sequencing

1.1 Illumina sequencing

1.1.1 Extraction of genome DNA

Total genome DNA from samples was extracted using CTAB method. DNA concentration and purity was monitored on 1% agarose gels. According to the concentration, DNA was diluted to 1ng/μL using sterile water.

1.1.2 Amplicon Generation

16S rRNA / 18S rRNA / ITS genes of distinct regions (16S V4/16S V3/16S V3-V4/16S V4-V5, 18S V4/18S V9, ITS1/ITS2, Arc V4) were amplified used specific primer (e.g. 16S V4: 515F- 806R, 18S V4: 528F-706R, 18S V9: 1380F-1510R, et.al.) with the barcode. All PCR reactions were carried out with 15 μL of Phusion® High -Fidelity PCR Master Mix (New England Biolabs); 2 μM of forward and reverse primers, and about 10 ng template DNA. Thermal cycling consisted of initial denaturation at 98 °C for 1 min, followed by 30 cycles of denaturation at 98 °C for 10 s, annealing at 50°C for 30 s, and elongation at 72°C for 30 s. Finally 72°C for 5 min.

Types	Amplified region	Fragment Length	Primers	Sequences (5'- 3')
Bacterial 16S	V4	300 bp	515F	GTGCCAGCMGCCGCGGTAA
			806R	GGACTACHVGGGTWTCTAAT
	V3-V4	470 bp	341F	CCTAYGGGRBGCASCAG
			806R	GGACTACNNGGGTATCTAAT
	V4-V5	450 bp	515F	GTGCCAGCMGCCGCGGTAA
			907R	CCGTCAATTCCTTTGAGTTT
	V5-V7 (for endophytic)	435 bp	799F	AACMGGATTAGATACCCKG
			1193R	ACGTCATCCCCACCTTCC
Archaeal 16S	V4-V5	400-500 bp	Arch519F	CAGCCGCCGCGGTAA
			Arch915R	GTGCTCCCCCGCCAATTCCT
18S	V4	350 bp	528F	GCGGTAATTCCAGCTCCAA
			706R	AATCCRAGAATTTCACCTCT
	V9	200 bp	1380F	CCCTGCCHTTTGTACACAC
			1510R	CCTTCYGCAGGTTCACCTAC
ITS	ITS1-1F	200-400 bp	ITS1-1F-F	CTTGGTCATTTAGAGGAAGTAA
			ITS1-1F-R	GCTGCGTTCTTCATCGATGC
	ITS1-5F		ITS5-1737F	GGAAGTAAAAGTCGTAACAAGG
			ITS2-2043R	GCTGCGTTCTTCATCGATGC
	ITS2	380 bp	ITS3-2024F	GCATCGATGAAGAACGCAGC
			ITS4-2409R	TCCTCCGCTTATTGATATGC
	ITS1-1F (for endophytic)	200-400 bp	ITS1-1F-F	CTTGGTCATTTAGAGGAAGTAA
			ITS1-1F-R	GCTGCGTTCTTCATCGATGC

1.1.3 PCR Products quantification and qualification

Mix same volume of 1X loading buffer (contained SYBR Green) with PCR products and operate electrophoresis on 2% agarose gel for detection. PCR products was mixed in equidensity ratios. Then, mixture PCR products was purified with Qiagen Gel Extraction Kit (Qiagen, Germany).

1.1.4 Library preparation and sequencing

Sequencing libraries were generated usingTruSeq® DNA PCR-Free Sample Preparation Kit (Illumina, USA) following manufacturer's recommendations and index codes were added. The library quality was assessed on the Qubit@ 2.0 Fluorometer (Thermo Scientific) and Agilent Bioanalyzer 2100 system. At last, the library was sequenced on an Illumina NovaSeq platform and 250 bp paired-end reads were generated.

1.2 MGI sequencing

1.2.1 Extraction of genome DNA

Genomic DNA of the samples is extracted using CretMag™ Power Soil DNA Kit (CretBiotech, China) according to manufacturer's instructions. The DNA extract was checked on 1% agarose gel, and DNA concentration and purity were determined with NanoDrop 2000 UV-vis spectrophotometer (Thermo Scientific, Wilmington, USA)

1.2.2 Amplicon Generation

Using the diluted genomic DNA as a template and based on the selected sequencing region, specific primers with barcodes, and ES Taq MasterMix (Dye) to prepare PCR mixtures. PCR reactions were performed in triplicate for each sample.

Primer regions include:

- 16S V4 region primers (515F and 806R): Identify bacterial diversity.
- 18S SSU (0817F and 1196R): Identify eukaryotic diversity.
- ITS1 region primers (ITS1F and ITS2): Identify fungal diversity.

Additionally, the amplified regions include: 16S V3-V4 / 16S V4-V5 / 16S V5-V7; Archaea 16S V4-V5 / Archaea 16S V8; 18S V9 and ITS2 regions.

1.2.3 PCR Products quantification and qualification

The PCR products of the same sample were mixed and then detected by 2% agarose gel electrophoresis. The qualified PCR products were purified using the AxyPrep DNA Gel Extraction Kit (Axygen Biosciences, Union City, CA, USA). After purification, the products were again verified by 2% agarose gel electrophoresis and quantified using a Quantus™ Fluorometer (Promega, USA).

1.2.4 Library preparation and sequencing

Library construction is carried out using the VAHTS® Universal Plus DNA Library Prep Kit for MGI V2 (Novizan, China). After quantification, sequencing was performed using MGI sequencing platform.

2 Data analysis

2.1 Paired-end reads assembly and quality control

2.1.1 Data split

Paired-end reads was assigned to samples based on their unique barcode and truncated by cutting off the barcode and primer sequence.

2.1.2 Data Filtration

Quality filtering on the raw tags were performed under specific filtering conditions to obtain the high-quality clean tags according to the fastp (https://github.com/OpenGene/fastp).

2.1.3 Sequence assembly

Paired-end reads were merged using FLASH (http://ccb.jhu.edu/software/FLASH/)(Magoc and Salzberg 2011), a very fast and accurate analysis tool, which was designed to merge paired-end reads when at least some of the reads overlap the read generated from the opposite end of the same DNA fragment.

2.1.4 Chimera removal

The tags were compared with the reference database (Silva database (16S/18S) https://www.arb-silva.de/; Unite Database(ITS), https://unite.ut.ee/) using UCHIME Algorithm (http://www.drive5.com/usearch/manual/uchime_algo.html) (Edgar et al. 2011) to detect chimera sequences, and then the chimera sequences were removed (Haas et al. 2011). Then the Effective Tags finally obtained.

2.2 OTU/ASV and Species annotation

2.2.1 OTU Clustering

Sequences analysis were performed by Uparse software (Uparse, http://drive5.com/uparse/) (Edgar 2013). Sequences with ≥97% similarity were assigned to the same OTUs. Representative sequence for each OTU was screened for further annotation.

2.2.2 ASV denoising

Amplicon sequence variant (ASV) were analysised by Deblur, which uses error profiles to obtain putative error-free sequences from second generation sequencing platform.

2.2.3 Species annotation

- 16S: For each representative sequence, the Silva Database (http://www.arb-silva.de/) (Quast et al. 2012) was used based on Mothur algorithm to annotate taxonomic information.
- 18S: For each representative sequence, the Silva Database (http://www.arb-silva.de/) (Quast et al. 2012) was used based on RDP classifier algorithm to annotate taxonomic information.

- ITS: For each representative sequence, the Unite Database (https://unite.ut.ee/) (Kõljalg et al. 2013) was used based on Mothur algorithm to annotate taxonomic information.

2.2.4 Phylogenetic relationship Construction

In order to study phylogenetic relationship of different OTUs, and the difference of the dominant species in different samples (groups), multiple sequence alignment were conducted using the MAFFT (https://mafft.cbrc.jp/alignment/software/) (Katoh et al. 2002).

2.2.5 Data Normalization

OTUs abundance information were normalized using a standard of sequence number corresponding to the sample with the least sequences. Subsequent analysis of alpha diversity and beta diversity were all performed basing on this output normalized data.

3 Alpha Diversity

Alpha diversity is applied in analyzing complexity of species diversity for a sample through 6 indices, including Observed-species, Chao1, Shannon, Simpson, ACE, Good-coverage. All this indices in our samples were calculated with QIIME and displayed with R software.

Two indices were selected to identify Community richness:

- Chao -the Chao1 estimator (http://www.mothur.org/wiki/Chao);
- ACE -the ACE estimator (http://www.mothur.org/wiki/Ace);

Two indices were used to identify Community diversity:

- Shannon - the Shannon index (http://www.mothur.org/wiki/Shannon);
- Simpson - the Simpson index (http://www.mothur.org/wiki/Simpson);

One indice to characterized Sequencing depth:

- Coverage - the Good's coverage (http://www.mothur.org/wiki/Coverage)

4 Beta Diversity

Beta diversity analysis was used to evaluate differences of samples in species complexity, Beta diversity on both weighted and unweighted unifrac were calculated by QIIME software.

Cluster analysis was preceded by principal component analysis (PCA), which was applied to reduce the dimension of the original variables using the stats package and ggplot2 package in R software.

Principal Coordinate Analysis (PCoA) was performed to get principal coordinates and visualize from complex, multidimensional data. A distance matrix of weighted or unweighted unifrac among samples obtained before was transformed to a new set of orthogonal axes, by which the maximum variation factor is demonstrated by first principal coordinate, and the second maximum one by the second principal coordinate, and so on. PCoA analysis was displayed by stats package and ggplot2 package in R software.

Unweighted Pair-group Method with Arithmetic Means (UPGMA) Clustering was performed as a type of hierarchical clustering method to interpret the distance matrix using average linkage and was conducted by QIIME software.

Reference

Edgar, Robert C. 2013. "UPARSE: Highly Accurate OTU Sequences from Microbial Amplicon Reads." *Nature Methods* 10 (10): 996–98. https://doi.org/10.1038/nmeth.2604.

Edgar, Robert C., Brian J. Haas, Jose C. Clemente, Christopher Quince, and Rob Knight. 2011. "UCHIME Improves Sensitivity and Speed of Chimera Detection." *Bioinformatics (Oxford, England)* 27 (16): 2194–2200. https://doi.org/10.1093/bioinformatics/btr381.

Haas, B. J., D. Gevers, A. M. Earl, M. Feldgarden, D. V. Ward, G. Giannoukos, D. Ciulla, et al. 2011. "Chimeric 16S rRNA Sequence Formation and Detection in Sanger and 454-Pyrosequenced PCR Amplicons." *Genome Research* 21 (3): 494–504. https://doi.org/10.1101/gr.112730.110.

Katoh, Kazutaka, Kazuharu Misawa, Kei-ichi Kuma, and Takashi Miyata. 2002. "MAFFT: A Novel Method for Rapid Multiple Sequence Alignment Based on Fast Fourier Transform." *Nucleic Acids Research* 30 (14): 3059–66. https://doi.org/10.1093/nar/gkf436.

Kõljalg, Urmas, R. Henrik Nilsson, Kessy Abarenkov, Leho Tedersoo, Andy F. S. Taylor, Mohammad Bahram, Scott T. Bates, et al. 2013. "Towards a Unified Paradigm for Sequence-Based Identification

of Fungi.” *Molecular Ecology* 22 (21): 5271–7. https://doi.org/10.1111/mec.12481.

Magoc, T., and S. L. Salzberg. 2011. “FLASH: Fast Length Adjustment of Short Reads to Improve Genome Assemblies.” *Bioinformatics* 27 (21): 2957–63. https://doi.org/10.1093/bioinformatics/btr507.

Quast, Christian, Elmar Pruesse, Pelin Yilmaz, Jan Gerken, Timmy Schweer, Pablo Yarza, Jörg Peplies, and Frank Oliver Glöckner. 2012. “The SILVA Ribosomal RNA Gene Database Project: Improved Data Processing and Web-Based Tools.” *Nucleic Acids Research* 41 (D1): D590–D596. https://doi.org/10.1093/nar/gks1219.
