# Supplementary figures and images for: Engineered probiotics platform for resolvin E1 biosynthesis confers protection against inflammatory disease
Source: Clin Transl Med. 2026 Jul 19;16(7):e70746. doi: 10.1002/ctm2.70746 (PMC13382362; doi:10.1002/ctm2.70746)

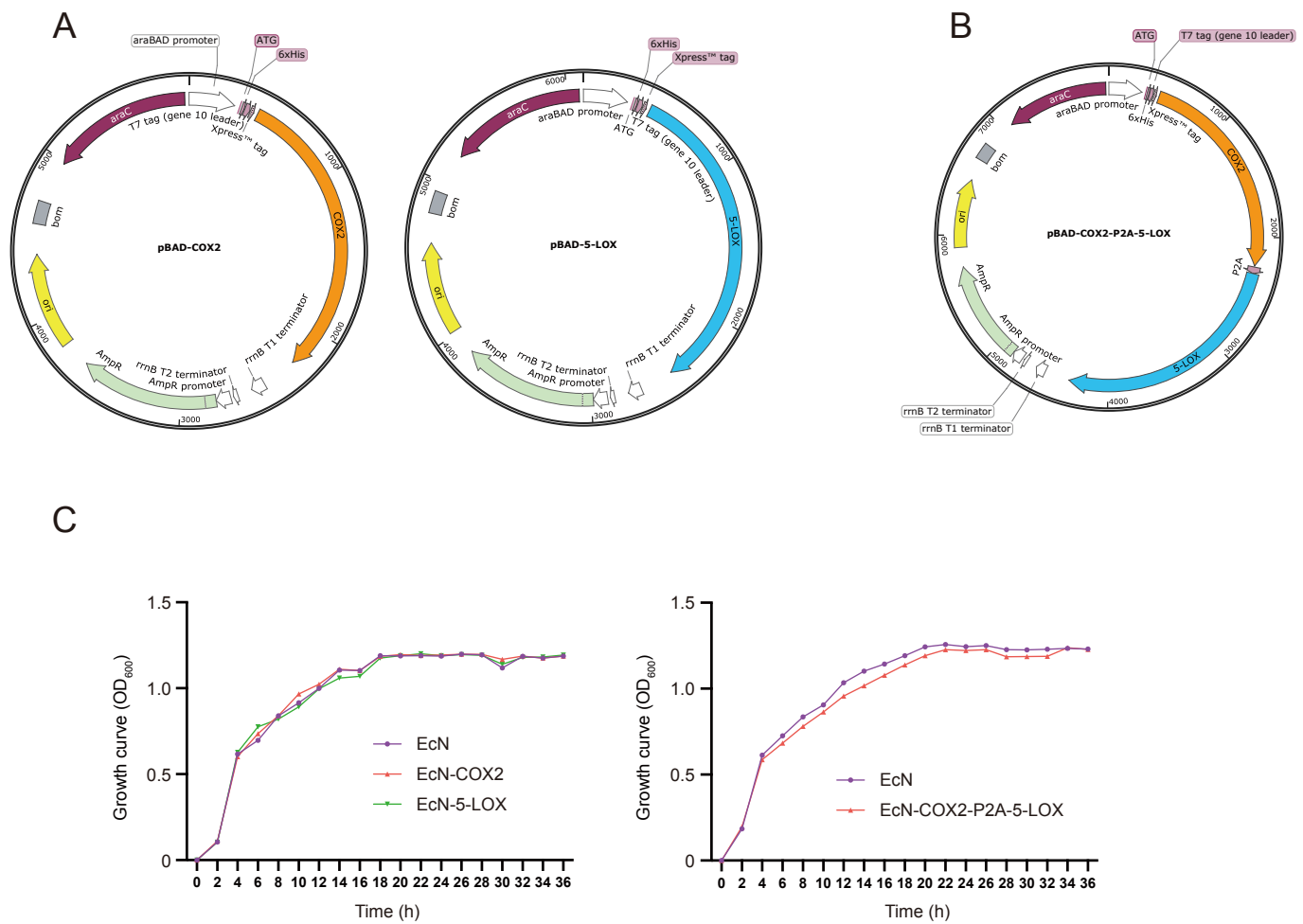

Supplement: Supplementary file 2 — Figure S1: Construction and characterization of recombinant expression vectors and engineered strains, related to Figure 1. (A and B) Plasmid profile of the recombinant arabinose‐inducible araBAD promoter containing pBAD expression vector for COX2 and 5‐LOX (A) or COX2‐P2A‐5‐LOX (B). (C) Growth curves of wild‐type EcN and recombinant strains. Left panel: Comparison of EcN, EcN‐COX2 and EcN‐5‐LOX. Right panel: Comparison of EcN and EcN‐COX2‐P2A‐5‐LOX. Bacterial growth was monitored by measuring optical density at 600 nm (OD600) over 36 h. [file CTM2-16-e70746-s002.pdf]

A

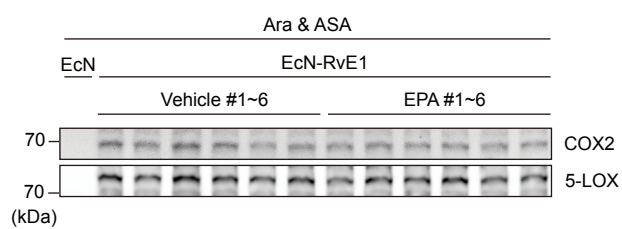

B

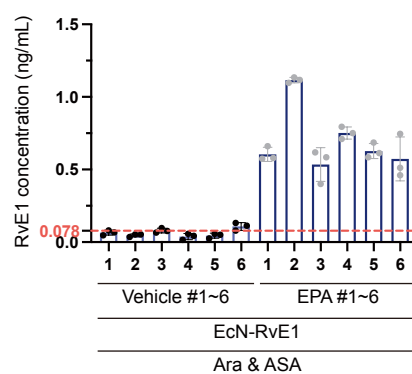

C

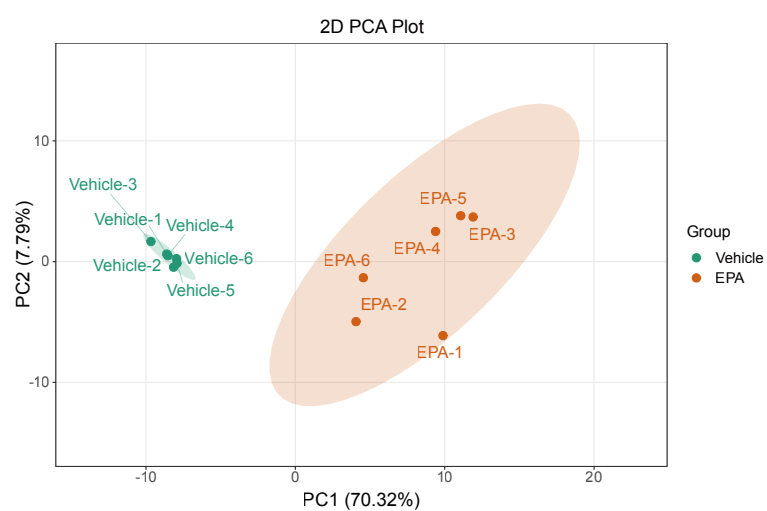

Supplement: Supplementary file 3 — Figure S2: Oxylipin analysis of EcN‐RvE1 culture supernatants by LC‐MS/MS, related to Figure 1. (A and B) Western blot analysis confirming the expression of COX2 and 5‐LOX (A) and RvE1 concentration in culture supernatants measured by ELISA (B) in EcN‐RvE1 sample for oxylipin analysis induced with arabinose (Ara) and ASA. The red dashed line and number indicate the limit of detection of the ELISA assay. The bars are represented as mean ± SD (n = 3 per group). (C) Principal component analysis (PCA) of oxylipin profiles detected by LC‐MS/MS. PC1 (first principal component) and PC2 (second principal component) are shown on the x‐ and y‐axes, respectively, with the percentages indicating the proportion of variance explained by each principal component (70.32% for PC1 and 17.79% for PC2). Each dot represents an individual sample, with samples from the same group coloured identically. [file CTM2-16-e70746-s007.pdf]

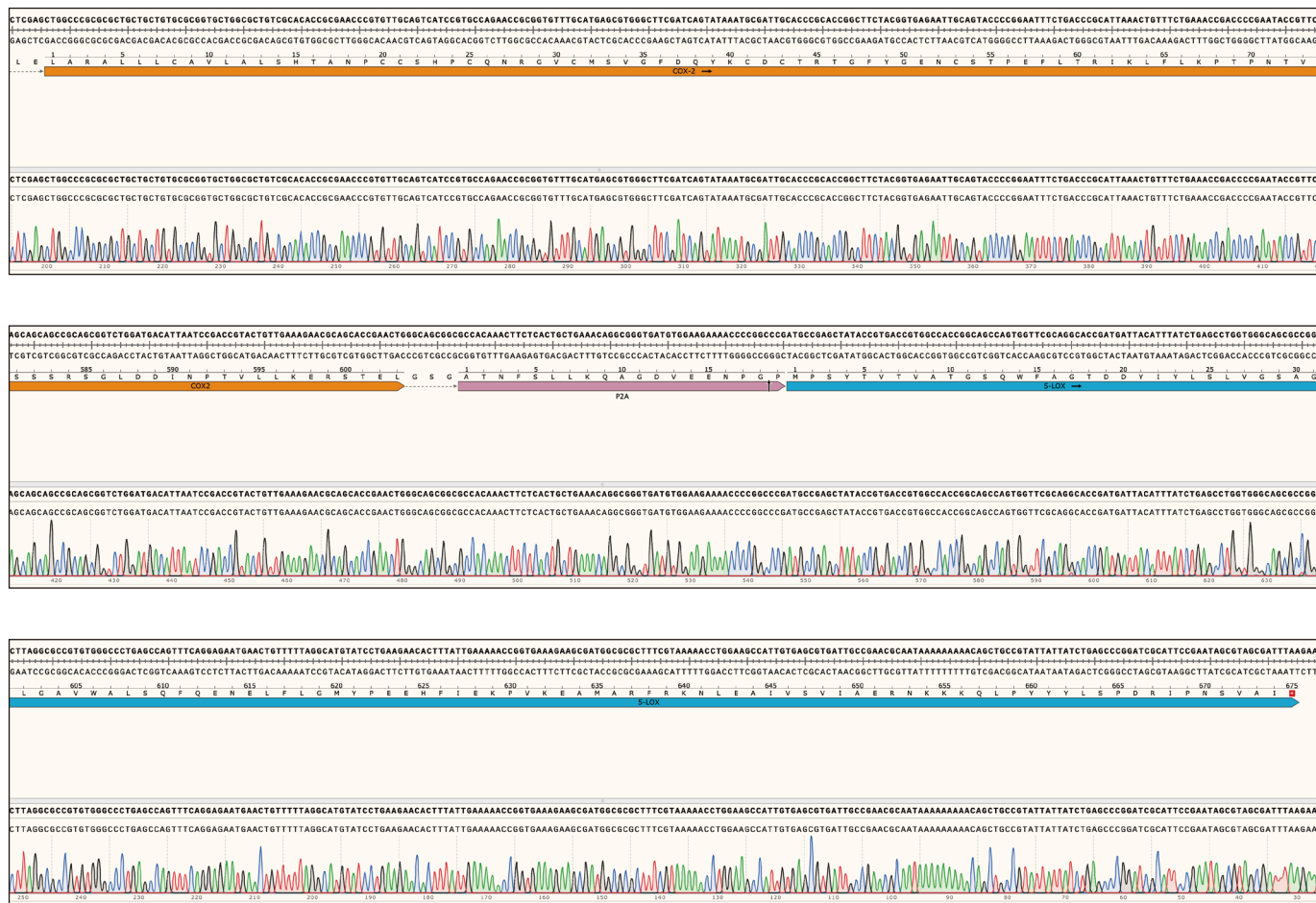

Supplement: Supplementary file 4 — Figure S3: Molecular confirmation of EcN‐RvE1 colonization by colony PCR, related to Figure 2. (A) Representative sequencing chromatogram of colony PCR products from randomly selected colonies grown from faecal samples collected at day 60 post‐gavage. PCR was performed using gene‐specific primers for COX2 and 5‐LOX. [file CTM2-16-e70746-s001.pdf]

A

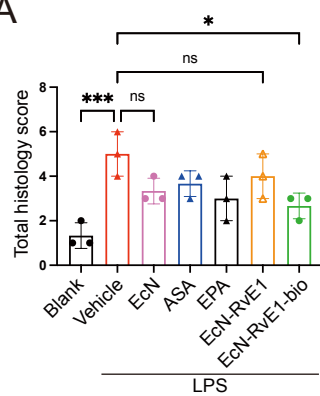

B

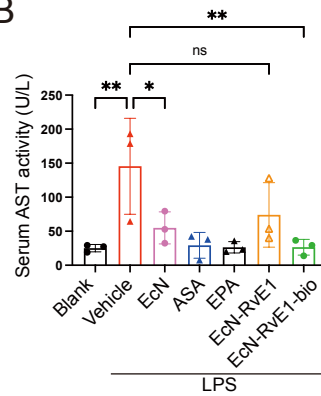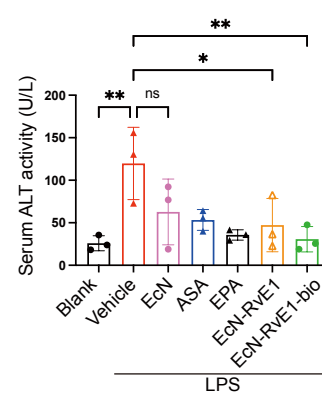

Supplement: Supplementary file 5 — Figure S4: Total histology score and serum liver enzyme activity levels in LPS‐induced acute inflammation model, related to Figure 3. (A) Total histological scores of small intestine tissues from each experimental group. Tissue sections were scored based on parameters including structural damage, inflammatory cell infiltration and mucosal integrity. The bars are represented as mean ± SD (n = 3 per group). Statistics: Ordinary one‐way ANOVA multiple comparisons tests (ns, not significant, * p < .05, ** p < .01, *** p < .001, **** p < .0001). (B) Serum activity levels of aspartate aminotransferase (AST) and alanine aminotransferase (ALT) measured in each group. The bars are represented as mean ± SD (n = 3 per group). Statistics: Ordinary one‐way ANOVA multiple comparisons tests (ns, not significant, * p < .05, ** p < .01, *** p < .001, **** p < .0001). [file CTM2-16-e70746-s011.pdf]

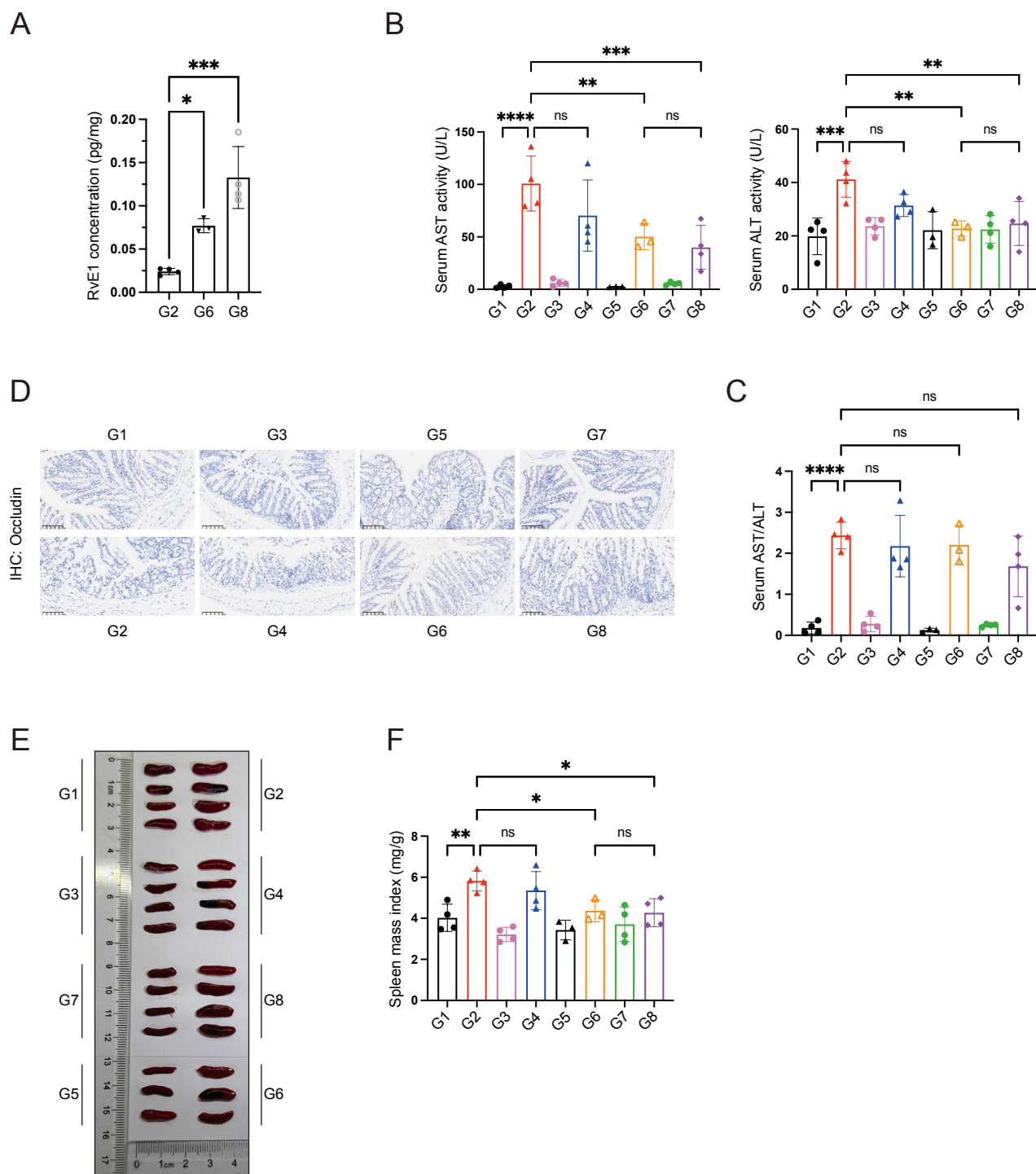

Supplement: Supplementary file 6 — Figure S5: Direct detection of RvE1 in colonic tissues, serum liver enzyme levels, tight junction protein expression, and spleen index in DSS‐induced colitis model, related to Figure 4. (A) RvE1 concentration in colonic tissue homogenates measured by ELISA in indicated experimental group. The bars are represented as mean ± SD (n = 3 or 4 per group). Statistics: Ordinary one‐way ANOVA multiple comparisons tests (ns, not significant, * p < .05, ** p < .01, *** p < .001, **** p < .0001). (B and C) Serum activity levels of AST and ALT (B) and AST/ALT ratio (C) measured in each experimental group. The bars are represented as mean ± SD (n = 3 or 4 per group). Statistics: Ordinary one‐way ANOVA multiple comparisons tests (ns, not significant, * p < .05, ** p < .01, *** p < .001, **** p < 0.0001). (D) Representative IHC images of the tight junction protein Occludin in colonic tissues from indicated groups. The scale bar indicates 100 µm. (E and F) Representative images of spleen morphology from each experimental group (E) and corresponding quantification of spleen mass index (F). The bars are represented as mean ± SD (n = 3 or 4 per group). Statistics: Ordinary one‐way ANOVA multiple comparisons tests (ns, not significant, * p < .05, ** p < .01, *** p < .001, **** p < .0001). [file CTM2-16-e70746-s006.pdf]

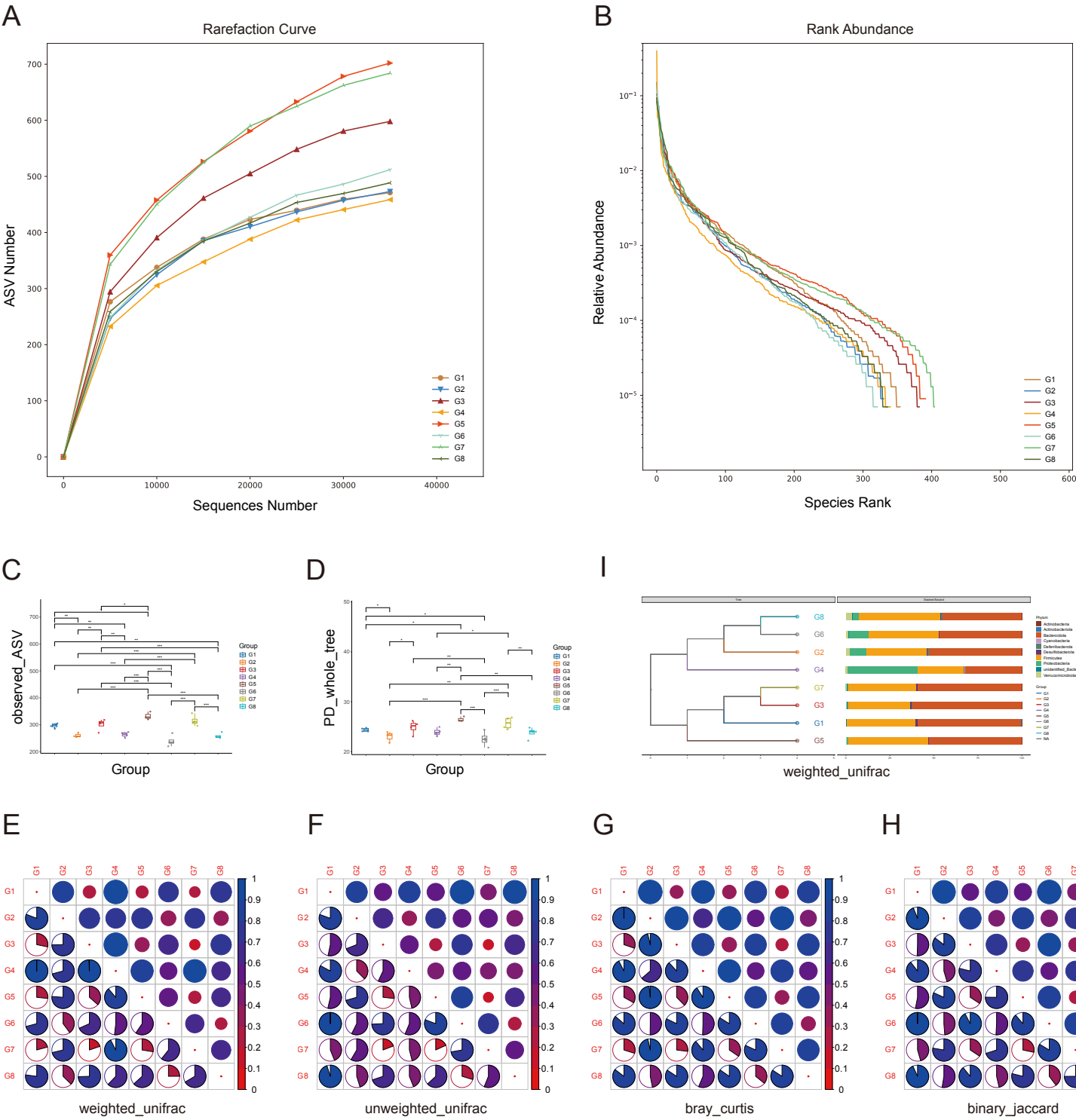

Supplement: Supplementary file 7 — Figure S6: Rarefaction curves, rank abundance and beta diversity analyses of gut microbiota in DSS‐induced colitis model, related to Figure 5. (A) Rarefaction curves of 16S rDNA amplicon sequencing data for all group samples. The curves show the number of ASVs (Amplicon Sequence Variants) as a function of sequencing reads. All curves plateaued after approximately 30 000 reads, indicating that sequencing depth was sufficient to capture the majority of microbial diversity. (B) Rank abundance curves showing species richness and evenness across experimental groups. The width of the curve reflects species richness, and the smoothness reflects species evenness. (C and D) Alpha diversity analysis shown as observed ASV count (C) and PD whole tree (phylogenetic diversity) index (D) across experimental groups. The observed ASV count directly reflects species richness, and the PD whole tree index measures the sum of branch lengths in the phylogenetic tree, reflecting the evolutionary diversity of the microbial community. Data are presented as box plots, with the horizontal line within each box indicating the median value (n = 3 or 4 per group). Statistics: Kruskal–Wallis rank test (* p < .05, ** p < .01, *** p < .001). (E–H) Distance matrix heatmap of beta diversity based on weighted UniFrac distances (E), unweighted UniFrac distances (F), Bray–Curtis distances (G) and binary Jaccard distances (H). In the upper triangle, circles represent pairwise beta diversity between groups, with smaller and redder circles indicating smaller distances and thus lower compositional dissimilarity. The lower triangle uses colour intensity and circle area to convey the same information. (I) UPGMA hierarchical clustering tree based on weighted UniFrac distances, integrated with phylum‐level relative abundance distributions. The clustering tree shows overall structural relatedness among samples from different groups. [file CTM2-16-e70746-s010.pdf]

A

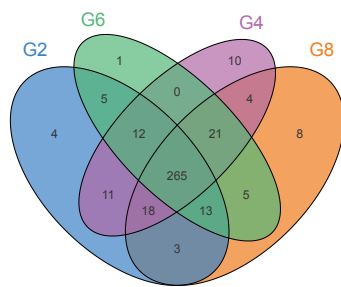

B

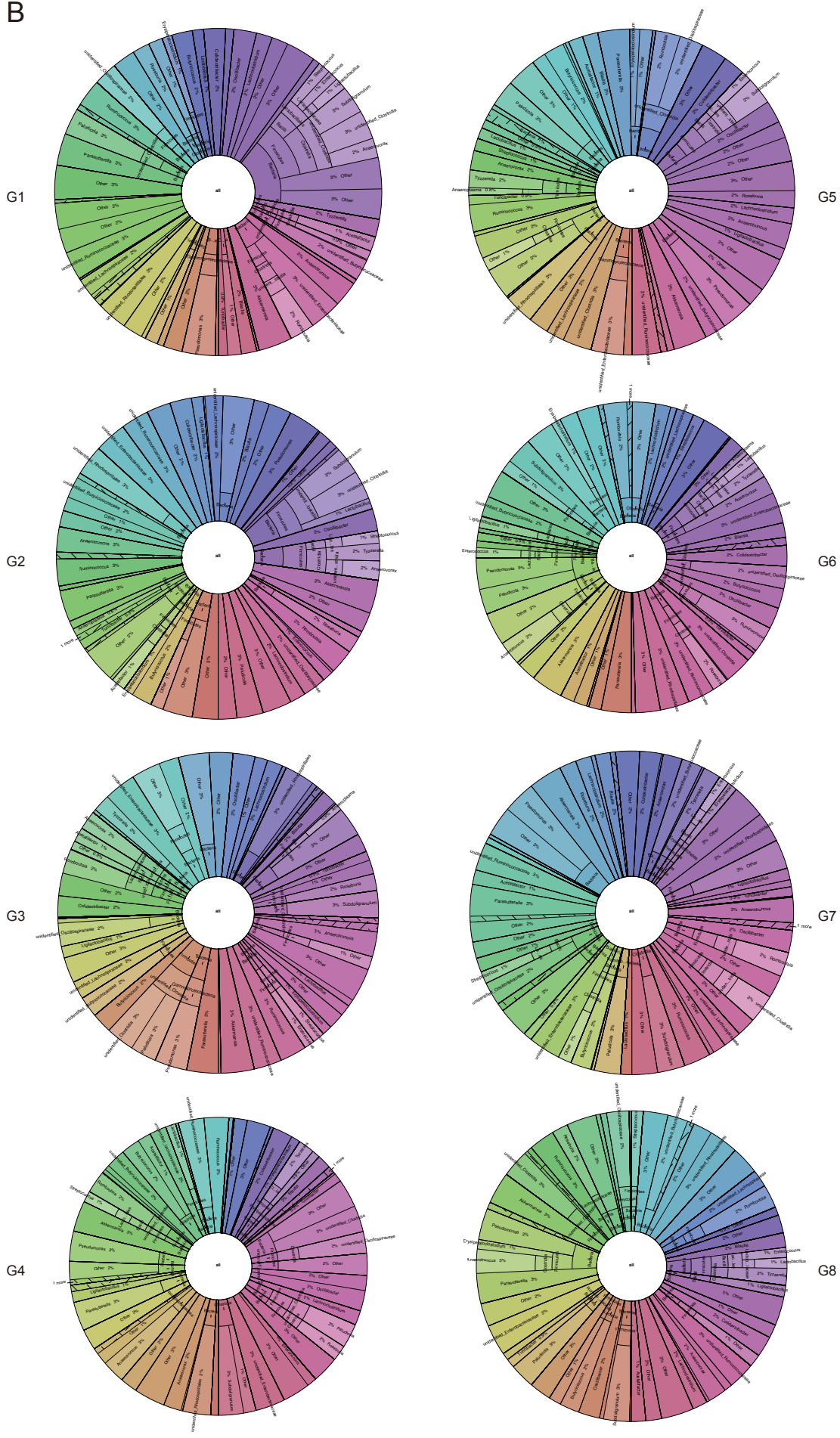

Supplement: Supplementary file 8 — Figure S7: ASV distribution and species annotation statistics, related to Figure 5. (A) Venn diagram showing the distribution of unique and shared ASVs among selected groups. Each circle represents a group, and overlapping regions indicate the number of ASVs shared between groups. The numbers in non‐overlapping regions represent group‐specific ASVs. (B) Species annotation statistics showing the number of taxa annotated at each taxonomic level (Phylum, Class, Order, Family, Genus, Species) for each sample group. The bar chart displays the count of identified taxa per group across classification levels, reflecting the depth of taxonomic annotation achieved. [file CTM2-16-e70746-s009.pdf]

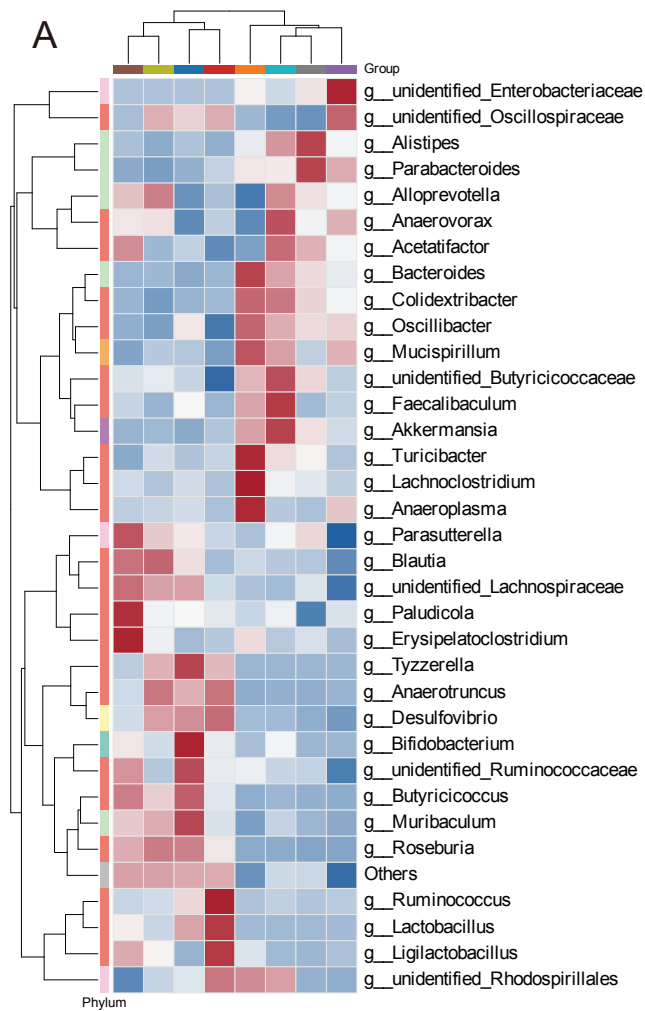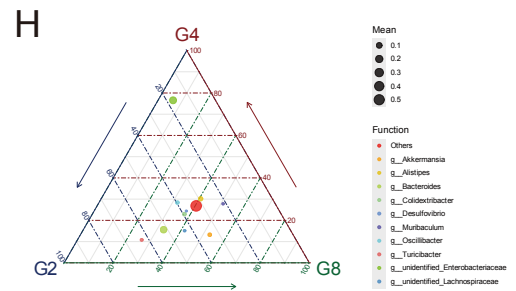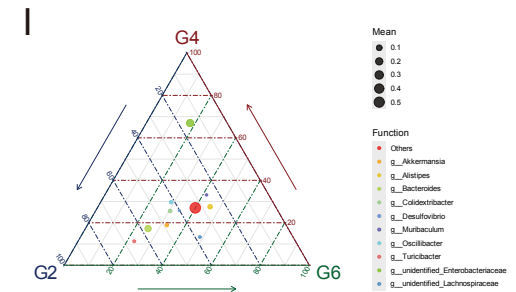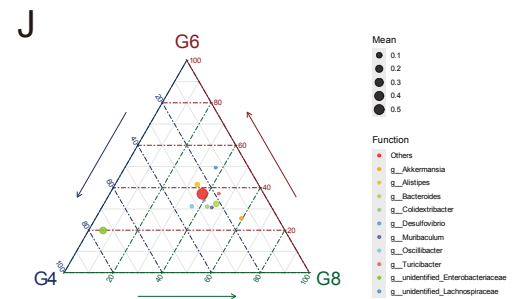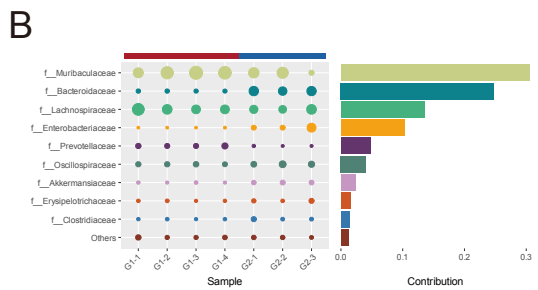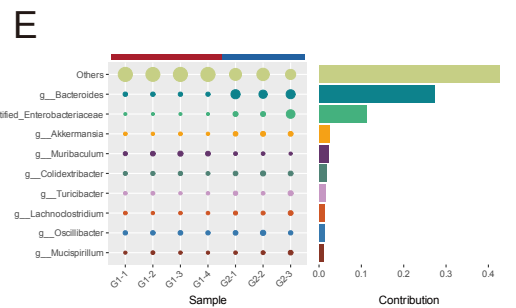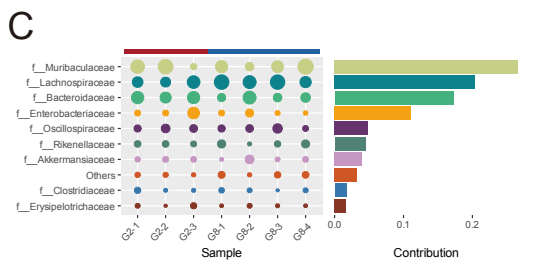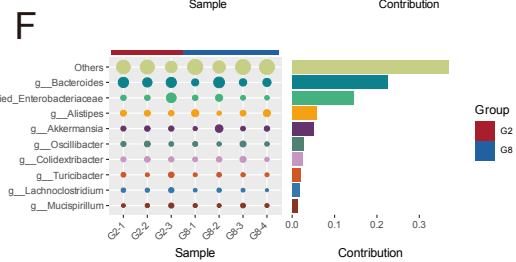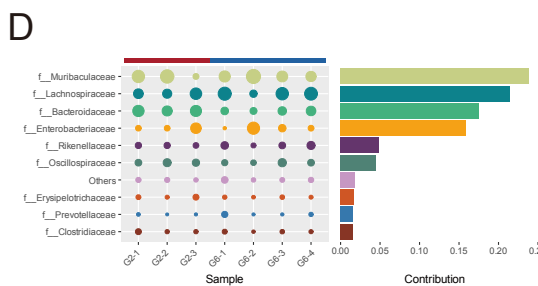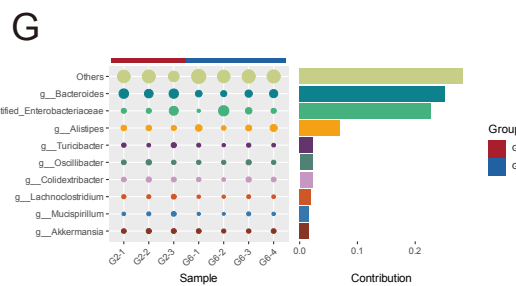

Supplement: Supplementary file 9 — Figure S8: Differential abundance and similarity percentage analysis of gut microbiota, related to Figure 5. (A) Heatmap showing the distribution of differentially abundant species at the genus level across experimental groups. The colour gradient represents Z‐score standardized relative abundance, with red indicating higher abundance and blue indicating lower abundance. Hierarchical clustering of both samples and genera is shown. (B–G) Similarity percentage (Simper) analysis showing the contribution of bacterial families (B–D) or genera (E–G) to the dissimilarity between G1 and G2 (B and E), G2 and G8 (C and F) and G2 and G6 (D and G). The bar chart displays the top contributing families or genera. (H–J) Ternary plot analysis at the genus level comparing the distribution of dominant species among three indicated groups. Each vertex represents a group, each circle represents a bacterial genus, and circle size is proportional to relative abundance. Circles positioned closer to a vertex indicate higher abundance in that group. [file CTM2-16-e70746-s008.pdf]
