# Supplementary material for: Engineered probiotics platform for resolvin E1 biosynthesis confers protection against inflammatory disease
Source: Clin Transl Med. 2026 Jul 19;16(7):e70746. doi: 10.1002/ctm2.70746 (PMC13382362; doi:10.1002/ctm2.70746)
Supplement: Supplementary file 10 — Supporting Information [file CTM2-16-e70746-s003.docx]

Table S1

The sequences of primers used in this study

| Name | Sequence (5’ ~ 3’) |
| --- | --- |
| Primers for the construction of pBAD vector expressing various genes | |
| pBAD-COX2-F | CAGCACCGAACTGTAAGAATTCGAAGCTTGGCTGTT |
| pBAD-COX2-R | TCTTACAGTTCGGTGCTGCGTTCTT |
| pBAD-5LOX-F | AGCTCGAGCCGAGCTATACCGTGACCGT |
| pBAD-5LOX-R | TATAGCTCGGCTCGAGCTCGGATCCCCA |
| pBAD-F | ATGCCATAGCATTTTTATCC |
| pBAD-R | GATTTAATCTGTATCAGG |
| seq-COX2-f | CTGCGTCGCAAATTCATCCC |
| seq-COX2-f2 | AAAGTGAGCCAGGCGAGCAT |
| seq-5LOX-f | TGCCGCGTGATATCCAGTTC |
| seq-5LOX-f2 | CCGAGCTATACCGTGACCGT |
| Primers for RT-qPCR on mouse cDNA | |
| ll1b-f | TCGCTCAGGGTCACAAGAAA |
| ll1b-r | CATCAGAGGCAAGGAGGAAAAC |
| Tnf-f | AGGGTCTGGGCCATAGAACT |
| Tnf-r | CCACCACGCTCTTCTGTCAC |
| ll6-f | ACGGCCTTCCCTACTTCACA |
| ll6-r | CATTTCCACGATTTCCCAGA |
| ll10-f | AGCCGGGAAGACAATAACTG |
| ll10-r | GGAGTCGGTTAGCAGTATGTTG |
| Actb-f | TGTCCACCTTCCAGCAGATGT |
| Actb-r | AGCTCAGTAACAGTCCGCCTAGA |

The coding sequences of COX2 and 5-LOX linked by P2A (codon-optimized for prokaryotic expression)

CTGGCCCGCGCGCTGCTGCTGTGCGCGGTGCTGGCGCTGTCGCACACCGCGAACCCGTGTTGCAGTCATCCGTGCCAGAACCGCGGTGTTTGCATGAGCGTGGGCTTCGATCAGTATAAATGCGATTGCACCCGCACCGGCTTCTACGGTGAGAATTGCAGTACCCCGGAATTTCTGACCCGCATTAAACTGTTTCTGAAACCGACCCCGAATACCGTTCATTATATTCTGACACATTTCAAAGGTTTCTGGAATGTTGTGAACAACATTCCGTTTCTGCGTAACGCCATTATGAGCTATGTTCTGACGTCGCGCAGCCATCTGATCGATAGCCCGCCGACCTATAACGCGGATTACGGCTACAAAAGTTGGGAAGCCTTTAGCAACCTGAGCTATTACACGCGCGCGCTGCCGCCGGTGCCGGATGATTGCCCGACCCCGCTGGGCGTGAAAGGCAAAAAACAGCTGCCGGATTCAAATGAAATTGTGGAAAAACTGCTGCTGCGTCGCAAATTCATCCCGGACCCGCAGGGCAGCAACATGATGTTTGCATTTTTTGCGCAACATTTTACCCATCAGTTTTTCAAAACCGATCACAAACGCGGCCCGGCCTTTACCAACGGTCTGGGCCACGGCGTCGATCTGAACCACATTTACGGCGAAACCCTGGCGCGTCAGCGTAAACTGCGCCTGTTTAAAGATGGCAAAATGAAATATCAGATCATTGATGGCGAAATGTACCCGCCGACGGTGAAAGATACCCAGGCCGAAATGATTTATCCGCCGCAGGTGCCGGAACATCTGCGTTTCGCCGTGGGCCAGGAAGTTTTTGGCCTGGTGCCGGGCCTGATGATGTATGCCACCATTTGGCTGCGTGAACACAATCGCGTGTGCGATGTGCTGAAACAGGAACACCCGGAATGGGGTGACGAACAGCTGTTTCAGACCAGCCGCCTGATTCTGATTGGTGAAACCATTAAAATTGTGATTGAGGACTACGTGCAGCATCTGTCTGGCTATCACTTTAAACTGAAATTTGATCCGGAACTGCTGTTTAATAAACAGTTTCAGTATCAGAACCGTATTGCGGCGGAATTTAATACCCTGTACCACTGGCATCCGCTGCTGCCGGATACCTTTCAGATTCATGATCAGAAATACAATTATCAGCAGTTTATTTACAACAACAGCATCCTGCTGGAACACGGCATTACCCAGTTTGTGGAAAGTTTTACCCGCCAGATTGCGGGCCGCGTTGCCGGCGGCCGTAATGTGCCGCCGGCGGTGCAGAAAGTGAGCCAGGCGAGCATTGATCAGTCGCGCCAAATGAAATATCAGAGCTTTAACGAATATCGCAAACGCTTTATGCTGAAACCGTATGAATCATTCGAAGAACTGACGGGCGAAAAAGAAATGTCGGCGGAACTCGAAGCGCTGTATGGCGATATTGACGCGGTGGAACTGTACCCGGCGCTGCTGGTCGAAAAACCGCGCCCAGATGCGATTTTTGGCGAAACCATGGTCGAAGTGGGTGCGCCGTTCAGCCTGAAAGGTCTGATGGGCAACGTGATTTGCAGCCCGGCCTATTGGAAACCGTCAACCTTTGGCGGTGAAGTCGGCTTTCAGATTATTAATACTGCGAGCATTCAGAGCCTGATTTGCAATAATGTTAAAGGCTGCCCGTTTACCTCATTTAGCGTTCCGGACCCGGAACTGATTAAAACCGTTACCATTAACGCGAGCAGCAGCCGCAGCGGTCTGGATGACATTAATCCGACCGTACTGTTGAAAGAACGCAGCACCGAACTGGGCAGCGGC*GCCACAAACTTCTCACTGCTGAAACAGGCGGGTGATGTGGAAGAAAACCCCGGCCCG*ATGCCGAGCTATACCGTGACCGTGGCCACCGGCAGCCAGTGGTTCGCAGGCACCGATGATTACATTTATCTGAGCCTGGTGGGCAGCGCCGGCTGCAGCGAAAAACATCTGTTGGATAAACCGTTCTACAACGATTTTGAACGCGGCGCCGTGGACAGCTATGATGTGACCGTGGATGAAGAACTGGGCGAAATTCAGCTGGTGCGCATTGAAAAACGCAAATATTGGTTAAACGACGATTGGTACCTGAAATATATTACACTGAAAACCCCGCATGGCGATTACATTGAGTTCCCGTGCTACCGCTGGATTACCGGCGATGTTGAGGTGGTGCTGCGCGATGGCCGCGCCAAACTGGCGCGTGACGATCAGATTCATATCCTGAAACAGCATCGTCGCAAAGAACTGGAAACCCGCCAGAAGCAGTACCGTTGGATGGAATGGAACCCGGGCTTTCCGCTGTCGATTGATGCCAAATGCCATAAAGATCTGCCGCGTGATATCCAGTTCGATTCAGAAAAAGGCGTGGATTTTGTGCTGAACTACAGCAAAGCCATGGAAAACCTGTTCATTAACCGCTTTATGCATATGTTCCAGAGCTCATGGAATGATTTCGCCGATTTTGAAAAAATTTTCGTGAAAATTAGCAATACCATTTCAGAACGCGTGATGAACCATTGGCAGGAAGATCTGATGTTCGGCTACCAGTTTCTGAACGGTTGCAATCCAGTGCTGATTCGCCGCTGCACTGAACTGCCGGAAAAACTGCCGGTGACGACCGAAATGGTGGAATGCTCTCTGGAGCGCCAGCTGTCATTGGAACAGGAAGTCCAGCAGGGCAATATTTTTATTGTTGATTTTGAACTGCTGGACGGCATTGATGCGAATAAAACCGATCCGTGTACCCTGCAGTTCCTGGCCGCGCCTATTTGCCTGCTGTATAAAAACCTGGCGAATAAAATTGTGCCGATCGCCATTCAGCTGAATCAGATTCCGGGCGATGAAAACCCGATCTTTCTGCCGAGCGATGCGAAGTACGATTGGCTGCTGGCCAAAATTTGGGTGCGCTCAAGTGATTTTCACGTGCACCAGACCATTACCCACCTGCTGCGTACCCATCTGGTGAGCGAAGTGTTTGGAATCGCCATGTATCGCCAGCTGCCGGCCGTGCATCCGATTTTTAAACTGCTGGTGGCCCACGTGCGCTTTACCATTGCGATCAACACCAAAGCACGTGAACAGCTGATCTGCGAATGTGGTCTGTTTGATAAAGCAAATGCGACCGGCGGCGGCGGTCATGTGCAGATGGTGCAGCGTGCCATGAAAGATCTGACCTATGCGAGCCTGTGCTTCCCTGAAGCGATTAAAGCCCGTGGCATGGAAAGCAAAGAAGATATTCCGTATTATTTTTATCGTGATGATGGCCTGCTGGTGTGGGAAGCCATTCGCACCTTTACCGCGGAAGTGGTCGATATTTACTACGAAGGCGATCAAGTGGTCGAAGAAGATCCGGAACTGCAGGATTTCGTTAACGATGTGTACGTCTACGGCATGCGTGGTCGCAAAAGCAGCGGTTTTCCGAAAAGCGTCAAATCGCGTGAACAGCTGTCGGAATATCTGACCGTGGTCATTTTTACCGCGTCGGCGCAACACGCGGCGGTTAATTTTGGTCAGTATGATTGGTGCAGCTGGATTCCGAATGCGCCGCCGACGATGCGCGCGCCGCCGCCGACCGCCAAAGGCGTGGTGACCATTGAACAGATTGTGGATACCTTACCGGATCGCGGTCGCAGCTGCTGGCACTTAGGCGCCGTGTGGGCCCTGAGCCAGTTTCAGGAGAATGAACTGTTTTTAGGCATGTATCCTGAAGAACACTTTATTGAAAAACCGGTGAAAGAAGCGATGGCGCGCTTTCGTAAAAACCTGGAAGCCATTGTGAGCGTGATTGCCGAACGCAATAAAAAAAAACAGCTGCCGTATTATTATCTGAGCCCGGATCGCATTCCGAATAGCGTAGCGATTTAA

The red color represents COX2 sequence

The blue color represents 5-LOX sequence

The italic represents P2A sequence
